# Pressure-Spun Fibrous Surgical Sutures for Localized Antibacterial Delivery: Development, Characterization, and In Vitro Evaluation

**DOI:** 10.1021/acsami.3c07956

**Published:** 2023-09-20

**Authors:** Esra Altun, Cem Bayram, Merve Gultekinoglu, Rupy Matharu, Angelo Delbusso, Shervanthi Homer-Vanniasinkam, Mohan Edirisinghe

**Affiliations:** † Department of Mechanical Engineering, University College London (UCL), Torrington Place, London WC1E 7JE, U.K.; ‡ Department of Nanotechnology and Nanomedicine, Graduate School of Science and Engineering, Hacettepe University, Ankara 06800, Turkey; § Department of Civil, Environmental and Geomatic Engineering, University College London, Gower Street, London WC1E 6BT, U.K.

**Keywords:** pressurized gyration, surgical site infection, antibacterial suture, engineering, healthcare

## Abstract

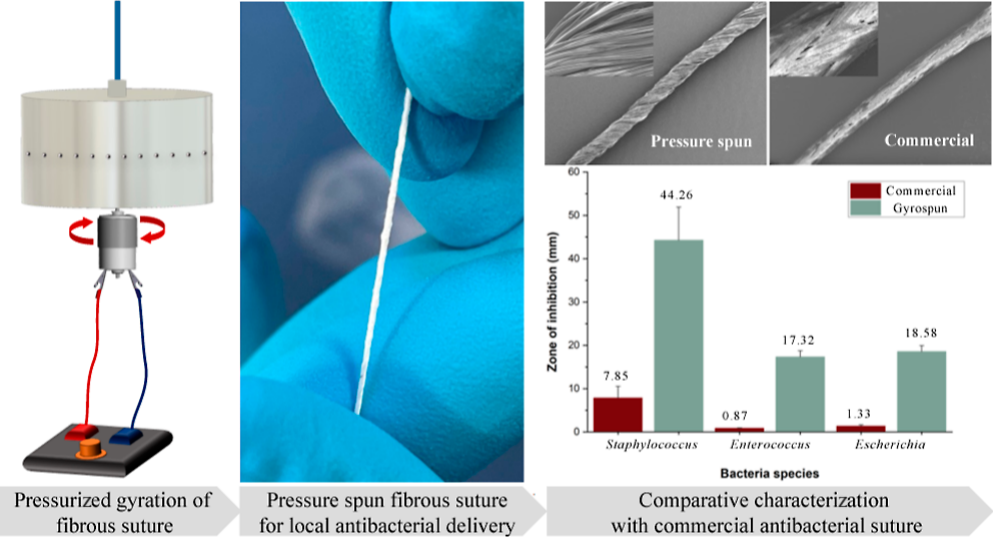

Surgical sutures designed to prevent infection are critical in addressing antibiotic-resistant pathogens that cause surgical site infections. Instead of antibiotics, alternative materials such as biocides have been assessed for coating commercially used sutures due to emerging antibiotic resistance concerns worldwide. This study has a new approach to the development of fibrous surgical sutures with the ability to deliver localized antibacterial agents. A new manufacturing process based on pressure spinning was used for the first time in the production of fibrous surgical sutures by physically blending antibacterial triclosan (Tri) agent with poly(lactic-*co*-glycolic acid) (PLGA) and poly(ethylene oxide) (PEO) polymers. Fibrous surgical sutures with virgin PLGA, virgin PEO, different ratios of PLGA–PEO, and different ratios of Tri-loaded PLGA–PEO fibrous sutures were produced to mimic the FDA- and NICE-approved PLGA-based sutures available in the market and compared for their characteristics. They were also tested simultaneously with commercially available sutures to compare their in vitro biodegradation, antibacterial, drug release, and cytotoxicity properties. After in vitro antibacterial testing for 24 h, sutures having 285 ± 12 μg/mg Tri loading were selected as a model for further testing as they exhibited antibacterial activity against all tested bacteria strains. The selected model of antibacterial fibrous sutures exhibited an initial burst of Tri release within 24 h, followed by a sustained release for the remaining time until the sutures completely degraded within 21 days. The cell viability assay showed that these surgical sutures had no cytotoxic effect on mammalian cells.

## 1 Introduction

Surgical site infections (SSIs) are the third most common hospital-acquired infections reported, accounting for approximately 16% of all these infections.^1^ SSIs pose threats to patients’ safety worldwide and increase the costs of care in all healthcare systems. The UK National Institute for Health and Care Excellence (NICE) estimates that 5% of patients undergoing a surgical procedure develop an SSI, costing The National Health Service (NHS) between £10,000 and £100,000 per patient nationally.^2^ They occur within 30 days of surgery when microorganisms, such as bacteria, enter the incision made by the surgeon in the skin to operate. Serious SSIs can affect subcutaneous tissues, organs, or implanted material, while superficial ones involve only the skin. Most SSIs can be treated with commonly prescribed antibiotics. Early detection, management, and control of SSIs are crucial to prevent excessive antibiotic use due to emerging antibiotic resistance.^3,4^

Surgical sutures have been used for many millennia in wound repair, providing support to the tissue during the healing period. They have extensive use in clinical disciplines, relying upon the requirement anywhere sutures are used. They can be produced as absorbable as well as nonabsorbable sutures with different materials. Absorbable sutures have gained interest as they can be absorbed or degraded in a patient’s body over time without the necessity of removal.^5^ A variety of polymers and copolymers including polyglycolic acid (PGA), polylactic acid (PLA), polydioxanone (PDS), and poly (lactic-*co*-glycolic acid) (PLGA) have increased demand due to their versatility for the synthesis of absorbable surgical sutures.^6^

The role of antimicrobial sutures is critical to address antimicrobial-resistant pathogens in preventing SSIs as microbes can bind to normal sutures and can be the potential source of a dangerous incision infection. Therefore, sutures designed to prevent infection are critical in addressing antibiotic-resistant pathogens that cause SSIs and the reduction of hospital costs and resources required to deal with readmitted patients. Instead of antibiotics, alternative materials such as metal-based nanoparticles and biocides have been assessed for coating commercially used sutures due to emerging antibiotic resistance concerns worldwide.^7,8^ The first commercially available antibacterial absorbable surgical Vicryl Plus suture (braided polyglactin 910, Ethicon–Johnson & Johnson) was coated with the antibacterial and antifungal biocide triclosan (Tri) and approved by the US Food and Drug Administration (FDA) in 2002 and NICE in 2020 for preventing SSIs. Based on clinical studies, they are expected to replace non-Tri absorbable sutures and can reduce the risk of SSIs in a broad population of patients undergoing surgery.^9^ During the last several decades, nano- to microrange fibers from polymeric biomaterials have found themselves a reputable place among biomedical applications, including drug delivery and wound dressing.^10–13^ Their large surface-to-volume ratio, controllable porosity, extracellular matrix structure resemblance, biocompatibility and biodegradability, and nontoxic nature make them attractive for various fields. These fibers are sensitive to the neighboring environment and can effectually deliver active agents to desired locations. Moreover, the release performance can be modulated by controlling morphological properties such as the diameter and alignment of fibers or the selection of polymeric materials used in manufacturing.^14^

The prior art of general engineering processes for transformation of neat polymeric biomaterials into fibrous surgical sutures is based on melt or wet spinning processes, such as melt extrusion or electrospinning.^15,16^ Pressurized gyration is a relatively new method used in the production of fibrous materials incorporating active pharmaceutical ingredients in a single step and has not been assessed to replace other production methods in surgical suture manufacturing.^17^ Compared to electrospinning, which requires high-voltage electric application for production, pressurized gyration offers new prospects by offering nozzle-free, cost-effective, mass production in rapid time without requiring a high-voltage electric application for fiber production which requires special infrastructure and can also be an extra source of degradation conducive to the incorporation of biologics. It also offers ease of production with a high level of control over fiber morphology to meet function, design, size, and scale-up requirements, which may be expected to lower overall manufacturing costs.^18^

PLGA has been a leading biodegradable polymer used in the production of commercially absorbable surgical sutures, as it provides excellent biocompatibility and mechanical properties.^19,20^ However, its biodegradation rate, high cost, and relatively hydrophobic nature can limit its use. Complete absorption of conventional PLGA sutures occurs through hydrolysis within months. This rate can be regulated by incorporating a hydrophilic polymer like poly (ethylene oxide) (PEO) while improving the mechanical properties of the product and can be left in the patient without the necessity for removal.^21^ Moreover, PLGA is an expensive polymer, and its combination with relatively inexpensive PEO could reduce the production cost of the final product which is favorable from an industrial point of view. Furthermore, the addition of PEO to the system could alter the release profile of active ingredients from fibrous suture systems.^22^ After active agents are released, the polymers would degrade into nontoxic components and leave the body.

Parikh et al. addressed ophthalmic infections by developing antibiotic-eluting sutures that incorporated the levofloxacin drug into polycaprolactone (PCL) through electrospinning.^23^ Given the global concern of antibiotic resistance, our study explored alternative antimicrobials such as Tri to enhance the efficacy against SSIs. This innovative approach involved successfully integrating Tri into novel fibrous absorbable antibacterial surgical sutures using pressurized gyration. Notably, this marks a significant milestone in the field, expanding its application across various surgical procedures and enabling localized delivery of the antibacterial agent. The materials used were model materials specifically chosen for the work based on commercial Vicryl products. Produced samples were also compared with those commercially available surgical sutures with various characterization tests.

## 2 Experimental Section

### 2.1 Materials

75:25 PLGA (1.0 dL/g) was purchased from Corbion (The Netherlands). PEO (*M*_w_ 200,000) and Tri were obtained from Sigma-Aldrich (UK). Commercial Vicryl (synthetic PLGA-based (polyglactin 910) absorbable braided suture, size 3–0), Vicryl Rapide (irradiated polyglactin 910 suture for rapid absorption, size 3–0), and Vicryl Plus (triclosan-coated polyglactin 910 suture, size 3–0) were purchased from Aston Pharma (UK). Chloroform (99.0%, Sigma-Aldrich, UK) was used as the solvent for solution preparation. All reagents used were of analytical grade and utilized as received.

### 2.2 Suture Formation

A 25% (w/v) PLGA solution and a 15% (w/v) PEO solution were individually prepared for the formation of surgical suture samples by dissolving the polymers in chloroform. Binary solutions of PLGA–PEO (PP) were prepared in chloroform to simultaneously dissolve both PLGA and PEO in weight ratios of 85:15, 70:30, 60:40, and 50:50. For antibacterial suture samples, various Tri concentrations as 10, 20, 30, and 40% (w/w of total polymer concentration) were added to preselected (after production and morphological characterization analyses) PP solution. All solutions were prepared in airtight vials and magnetically stirred for 24 h at ambient temperature (23 ± 1 °C) before being subjected to pressurized gyration.

The physical properties of solutions provide information on viscoelastic properties and have significant control over the jet generation and structural form during the pressurized gyration process. Viscosity was measured using a programmable rheometer (DV-III Ultra, Brookfield Engineering Laboratories Inc., Massachusetts, USA). Surface tension was measured using a digital tensiometer (K9, Kruss GmbH, Germany) using the du Noüy ring method. All equipment was calibrated before testing, and each measurement was repeated three times to find the mean and standard deviation (SD). Table 1 indicates the abbreviations of samples and the viscosity and surface tension values of solutions used in the experiments.

**Table 1:** Measured Viscosity and Surface Tension Values for the PP Binary Blends

sample abbreviation	sample (w/w)	rheological properties	
		viscosity (mPa·s)	surface tension (mN/m)
V-PLGA	100:0 PLGA–PEO	2731 ± 1	37.3 ± 0.5
85:15 PP	85:15 PLGA–PEO	3062 ± 3	39.2 ± 0.3
70:30 PP	70:30 PLGA–PEO	3325 ± 4	41.9 ± 0.7
60:40 PP	60:40 PLGA–PEO	3572 ± 4	43.2 ± 0.5
50:50 PP	50:50 PLGA–PEO	3928 ± 3	45.4 ± 0.6
V-PEO	0:100 PLGA–PEO	2184 ± 6	43.3 ± 1.1
5 TPP	5% Tri–PLGA–PEO	3301 ± 5	42.3 ± 0.2
10 TPP	10% Tri–PLGA–PEO	3253 ± 4	45.3 ± 0.4
20 TPP	20% Tri–PLGA–PEO	3092 ± 5	46.5 ± 0.3
30 TPP	30% Tri–PLGA–PEO	2913 ± 3	47.2 ± 0.3
40 TPP	40% Tri–PLGA–PEO	2882 ± 6	49.1 ± 0.2

**Figure 1:**
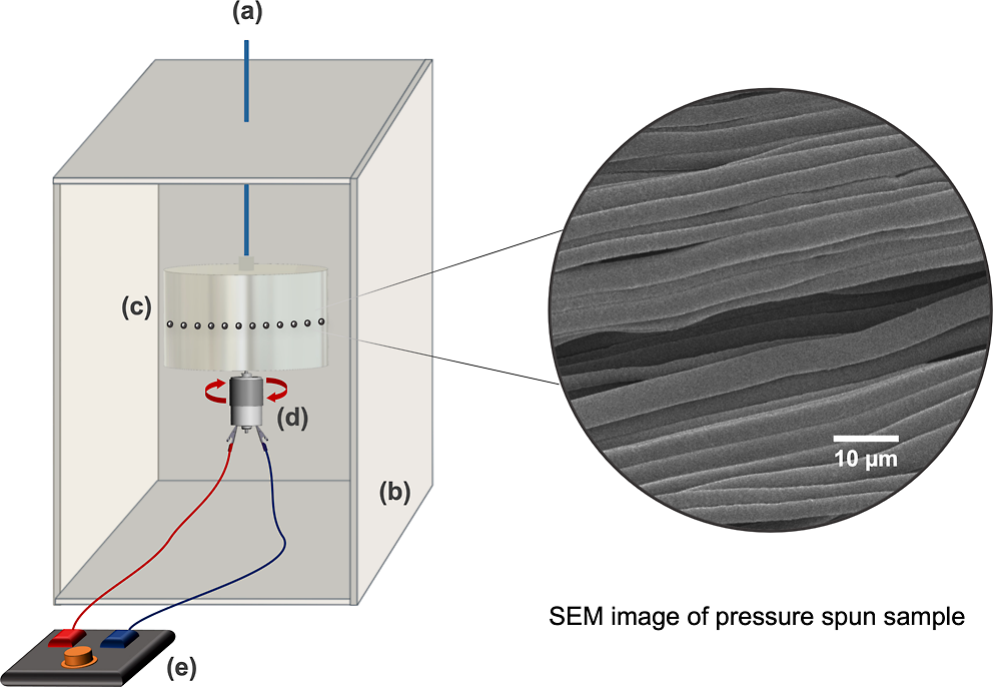
Schematic overview of the pressurized gyration equipment with a gas inlet that allows imparting N_2_ pressure to the system (up to 0.3 MPa) (a), a collection box that has a 100 mm distance with the rotary vessel for fiber collection (b), a rotating aluminum vessel (35 mm × 60 mm) with perforations (0.5 mm) for fiber production (c), a high-speed DC motor (d), and a speed controller (e) to maintain the high-speed and an SEM image of a pressure-spun sample on the RHS.

The suture manufacturing equipment (Figure 1) consists of a rotary aluminum cylindrical vessel, having 20 ∼0.5 mm diameter round perforations on the surface and contained in a box to collect the polymeric fibers conveniently. The top end of the rotary vessel is connected to the dinitrogen (N_2_) gas supply with a rotary joint. The bottom of the rotary vessel is attached to a DC motor that could produce high speeds through the process. The method is based on the manipulation of the Rayleigh–Taylor instability of a polymer solution and involves the formation of a polymer jet by the combined effects of centrifugal force, gas pressure, and high rotational speed.^24^ The polymer solution is forced through perforations in a rotating vessel, where it undergoes stretching and alignment due to centrifugal forces and air drag. As the solvent evaporates, solidified polymeric fibers form and deposit on the collection box walls. The alignment of the fibers is influenced by the interplay of various factors. The applied gas pressure controls the flow of the solution, directing it through the perforations. The combination of pressure, rotation, and physical properties of the material affects the alignment. Centrifugal forces generated by the rotation stretch align the fibers, while air drag assists in their orientation. Flow dynamics induced by pressure and rotation contribute to the alignment process, promoting a more ordered arrangement of the fibers. The degree of fiber alignment can be controlled by adjusting parameters such as N_2_ pressure, rotation speed, and the distance between the perforations and the collection area. This precise control enables the achievement of a high level of fiber alignment during the pressurized gyration process.^25^ The alignment of fibers is advantageous as it enhances mechanical properties such as strength and flexibility and can also influence properties like sustained release characteristics.

The properties of the polymer solution, including the molecular weight of the polymer, viscosity, surface tension, and solvent volatility, along with process parameters such as applied pressure and rotational speed, and system parameters like the size of the perforations and collection distance, as well as ambient conditions such as temperature and relative humidity, all have an impact on the morphology of the fibers produced.^26^ These parameters also affect the homogeneity and yield of the final product, necessitating careful optimization through experimentation for consistent and scaled-up manufacturing.

**Figure 2:**
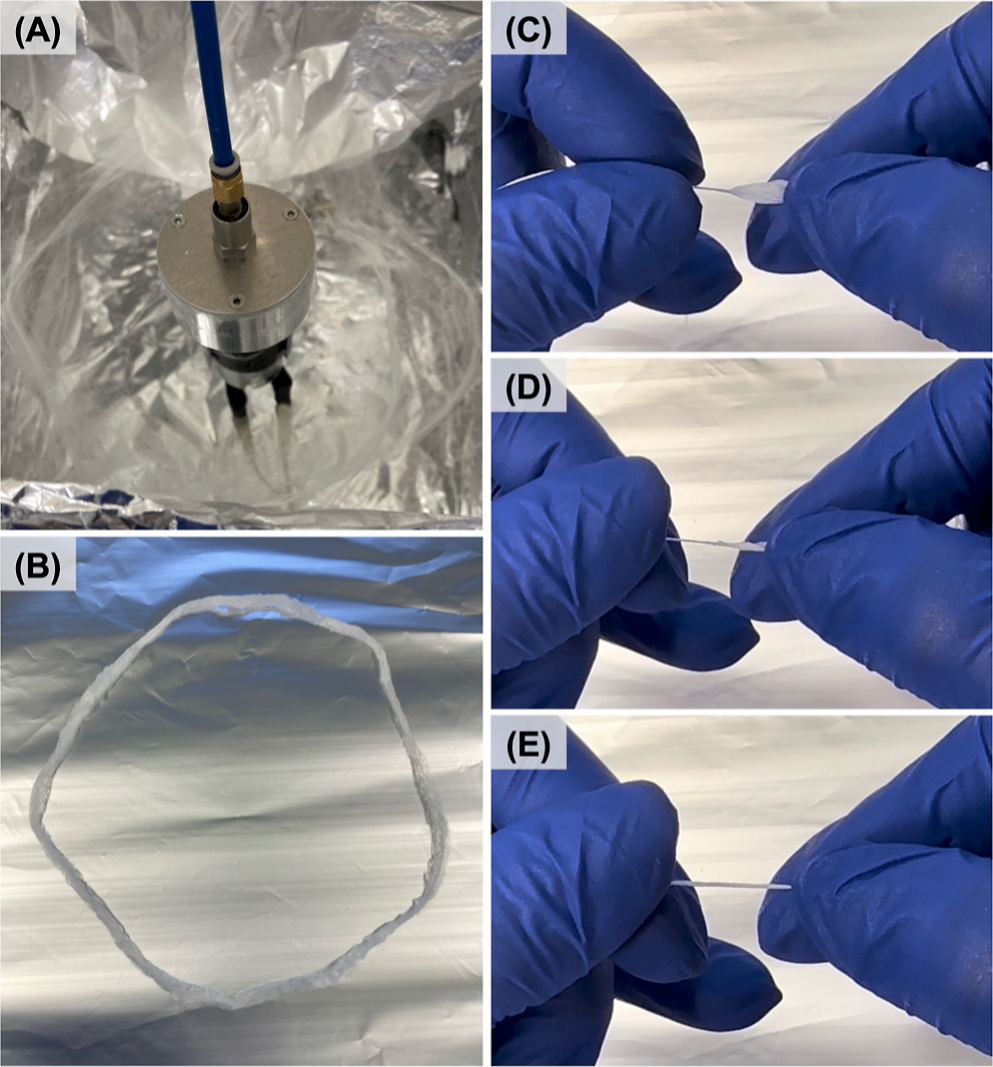
Images depicting the fibrous mesh at different stages of the fabrication process: (A) fibrous mesh immediately after the pressurized gyration, (B) collected fibrous mesh, and (C–E) twisting process to shape the fibrous mesh into a fibrous surgical suture.

After test runs, 1.5 mL of each prepared solution was placed inside the pressurized gyration vessel and spun at maximum rotational speed (10,000 rpm) with an applied gas pressure of 0.1 MPa (Figure 2A). The samples were then collected (Figure 2B), carefully twisted (Z-twists, ∼51 twists per inch (tpi), Figure 2C–E) to create 3–0 fibrous sutures for in vitro comparison with commonly used size 3–0 Vicryl Plus sutures in general soft tissue approximation and/or ligation.^27^ Nevertheless, it is important to highlight the versatility of pressurized gyration (pressure spinning), which allows for the exploration of fabricating sutures of different sizes to meet specific surgical requirements. Produced pressure-spun fibrous sutures were then placed into standard-sized Petri dishes in a vacuum oven overnight to ensure complete evaporation of chloroform and kept for further testing.

The twisting employed in this work has proven to be highly convenient, enabling the successful production of pressure-spun fibrous sutures with desirable characteristics including ease of handling. This approach not only ensures the practicality and convenience of the fibrous sutures but also emphasizes the potential for tailoring their properties to various surgical applications. To maintain the twisted state of the sutures, no additional external treatment or fixation method was employed. However, it is important to note that the inherent properties of the fibrous mesh, such as its natural shape memory and mechanical characteristics, contributed to the sutures’ ability to hold themselves in the twisted configuration.

### 2.3 Characterization

The production yield (%) was determined by measuring the mass of the pressure-spun samples obtained after the pressurized gyration process, using the following equation:
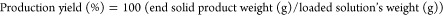
1

The morphology of produced samples was investigated by scanning electron microscopy (SEM, Hitachi S-3400n, Japan) using an accelerating voltage of 5 kV. Before imaging, samples were gold coated (Q150R ES, Quorum Technologies, UK) for 90 s. Fiber diameters were measured directly from SEM images using computer-aided image visualization software (Fiji). The obtained data were statistically analyzed, maximum, minimum, average, and SD values were calculated, and fiber diameter frequency graphics were prepared using the Origin Pro statistics software.

Drug loading (%) and encapsulation efficiency (%) of produced Tri-loaded fibrous sutures were measured by determining the total amount of Tri in the samples. In brief, the samples were completely dissolved in chloroform and the Tri content in the solution was analyzed by using a UV Spectrophotometer (Jenway 6305, Bibby Scientific, UK) at 282 nm. Equations 2 and 3 were used in the calculations with the aid of a preobtained calibration curve:
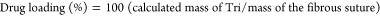
2
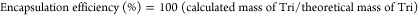
3

Fourier transform infrared spectroscopy (Nicolet iS50 FTIR Spectrometer, Thermo Fisher Scientific, UK) was used to analyze interactions between counterparts of the produced pressure-spun fibrous suture samples.

Hydrolytic degradation of commercial and pressure-spun fibrous sutures was performed in PBS (pH 7.4, Sigma-Aldrich, UK) at a constant temperature of 37 °C until complete degradation. Weighed samples (*W*_0_, 10 mg) were placed in amber vials containing 20 mL of a PBS buffer solution. After a certain noted time, the samples were taken from the vials, washed with distilled water, dried under vacuum at ambient temperature (23 °C) for 24 h, and weighed (*W*_f_). The method was also used for FTIR and mechanical analysis in the next stage of characterization tests. The weight loss (%) was calculated with eq 4
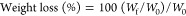
4

The surface wettability of pressure-spun fibrous surgical sutures was measured by an optical tensiometer (Attension Theta, Biolin Scientific, USA) using the meniscus method.^28^ Briefly, the fibrous samples were fixed on the tip of the dispenser and immersed in a liquid phase (deionized water). Then, the samples were withdrawn from the liquid at a speed of 1 mm/s, and the contact angle formed between the liquid/air interphase, and the suture was measured with high-speed camera images.

Thermal properties of gyrospun fibrous suture samples were tested via thermogravimetric analysis (TGA) and differential scanning calorimetric (DSC) in a simultaneous thermal analyzer (TA Instruments Q600-SDT, USA). TGA was used to monitor the mass of samples produced as a function of temperature by subjecting them to a controlled temperature program, while DSC was used to identify the heat energy uptake of samples tested within a regulated increase or decrease in temperature. In brief, the fibrous sutures were cut into pieces (<10 mg), placed in platinum pans inside a furnace, and subsequently subjected to high temperatures up to 600 °C at a heating rate of 10 °C/min under a nitrogen atmosphere. The change in mass and heat flow against temperature were recorded.

For mechanical characterization, tensile tests were applied to the produced fibrous suture samples and commercial Vicryl Normal, Vicryl Rapide, and Vicryl Plus sutures to measure the response of fibers to stress. To assess the knotted suture’s mechanical performance, all sutures were knotted using a square knot to secure the suture’s attachment. Ultimate tensile strength (UTS) calculations were performed in a specially designed lab-built setup. In the test procedure, pressure-spun fibrous sutures were secured in rubber clamps, one of which was fixed at the top and the other attached to a load carrier. The maximum force that the fibrous suture will withstand was determined by adding 10 g incremental weights to the lower carrier. UTS values were calculated according to the equation.

### 2.4 In Vitro Antibacterial Activity

The antibacterial properties of pressure-spun TPP fibrous surgical sutures and commercial Vicryl Plus sutures against *Staphylococcus aureus* (ATCC 6538P), vancomycin-resistant *Enterococcus faecalis* (VRE), and *Escherichia coli* (ATCC 11775) were determined with an in vitro agar diffusion test. A single colony of the chosen bacterial strain was suspended in 1000 μL of sterile deionized water and vortexed for 1 min. Hundred microliters of this suspension were spread onto a tryptic soy agar (TSA) plate. A 10 mm section of the fiber sample was placed in the center of the plate, ensuring the fiber was flat and in contact with the agar plate. 3.1 mg of pure Tri was used for the positive control. Following incubation of the plates at 37 °C for 24 h, the zone of inhibition was measured.

### 2.5 In Vitro Drug Release Study

The release studies were performed with UV spectroscopy at 282 nm to assess the release of Tri from a pressure-spun 40 TPP fibrous suture. Samples weighing 10 mg were placed in a sealed amber vial containing 20 mL of release medium (PBS, pH 7.4) and incubated on a shaker at a constant temperature of 37 °C. At predetermined time intervals, 3 mL of release medium was taken out, and 3 mL of fresh buffer solution was added to maintain an equal total solution volume. Prior to measuring, supernatants were filtered through a 0.45 μm Millipore filter to eliminate the impact of degraded polymers on the UV readings. The standard calibration curve was employed to determine the cumulative percentage of drug release, and release profiles were analyzed using Origin Pro software. Moreover, the cumulative amount of released Tri (μg) from the pressure-spun 40 TPP fibrous suture was calculated from the release profile obtained using the eq 5
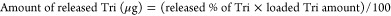
5

### 2.6 In Vitro Cytotoxicity Assay

The ISO 10993-5 standard was followed to conduct the cytotoxicity assay using the L929 mouse fibroblast cell line. Pressure-spun fiber suture samples were washed twice with PBS, immersed in 70% ethanol, and subjected to UV irradiation for sterilization. Subsequently, the samples were dried and placed in sealed vials containing 1 mL of culture media for a period of 72 h. The cell culture was carried out at 37 °C, >90% humidity, and 5% atmospheric CO_2_ using Dulbecco’s modified Eagle’s medium (DMEM, 90% (v/v)) supplemented with fetal bovine serum (FBS, 10% (v/v)). After incubation for 3 days, cells were seeded into a 96-well plate with a cell count of 1 × 10^4^ per well, and the extracted cell media were allowed to interact with the cells in the well plate for 24 h. A positive control of 10% dimethyl sulfoxide (DMSO) containing cell medium and a negative control of noninteracted DMEM–FBS medium were used. At the end of the assay, L929 cells were incubated with a 10% (4,5-dimethylthiazol-2-yl)-2,5-diphenyltetrazolium bromide (MTT) solution for 3.5 h at 37 °C. MTT crystals were dissolved with DMSO, and cell viability percentages were determined by the ELISA microplate reader at an absorbance of 570 nm.

### 2.7 Statistical Analysis

All the tests were carried out in triplicate (*n* = 3), and error bars represent SD. All the values were reported as mean ± SD *t*-test was used to calculate statistical significance.

## 3 Results and Discussion

### 3.1 Fibrous Suture Preparation

**Figure 3:**
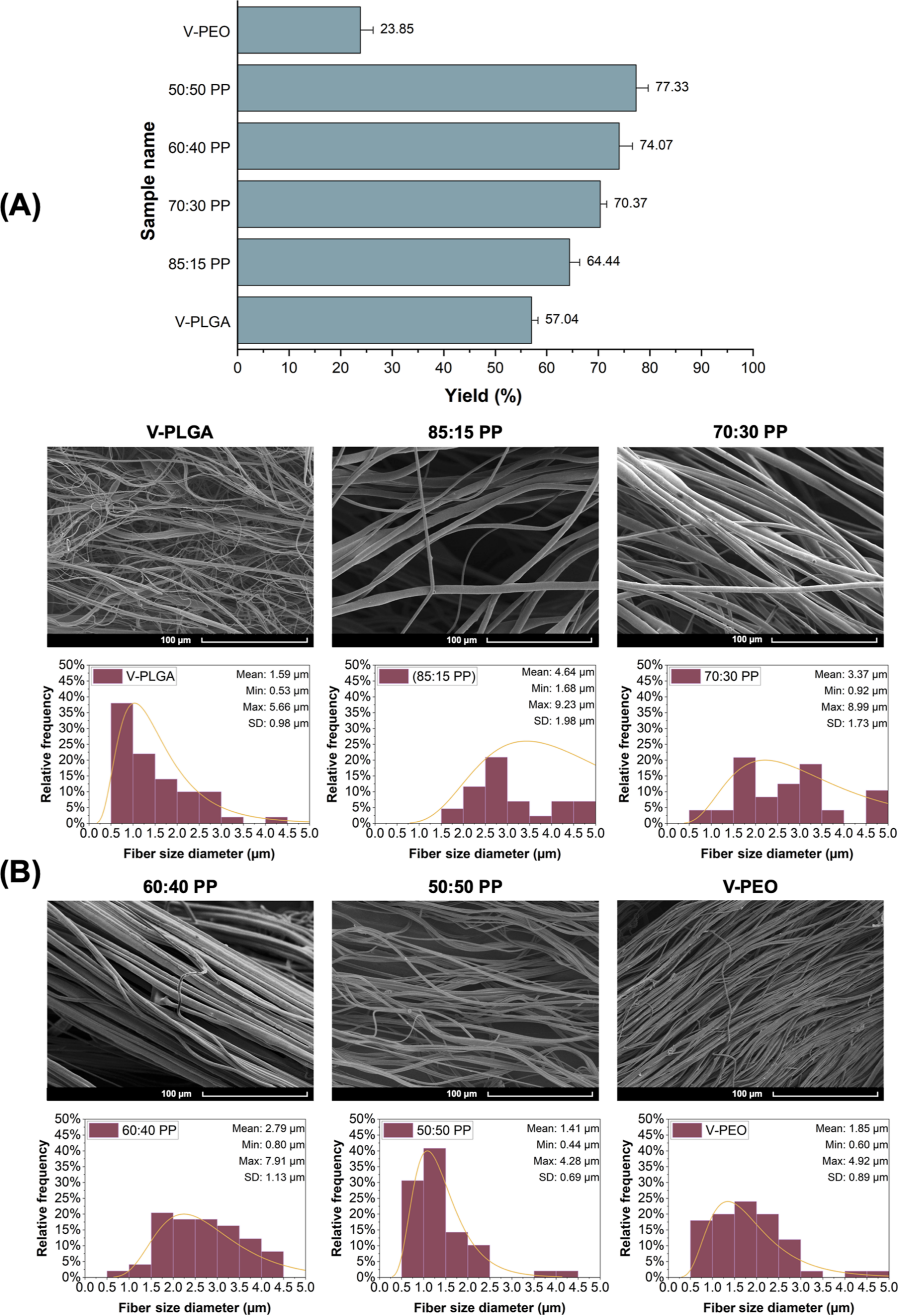
(A) Production yield (%) of different PP binary blends. (B) SEM images of pressure-spun fibrous suture samples with their corresponding diameter distribution histograms.

Before Tri was introduced into the binary system, V-PLGA, V-PEO, and different blends of PP between 85:15 (w/w) and 50:50 (w/w) were produced using pressurized gyration. Combining the two polymers together increased the production yield (%) for all PP binary systems compared to V-PLGA and V-PEO samples (Figure 3A).

Figure 3B displays SEM images and corresponding diameter distribution histograms of pressure-spun V-PLGA, V-PEO, and different blends of PP fibrous sutures. All samples displayed microfibers with fine and bead-free morphology, indicating a well-controlled spinning process.^29^ Also, microfibers displayed a more uniform distribution with the increasing PEO content in the PP system. The increased uniformity can be attributed to the improved compatibility between PEO and PP, resulting in better polymer flow and fiber formation during the spinning process as observed by Evrova et al.^30^ The mean diameter of PP microfibers decreased with increasing PEO content, starting at 4.64 ± 1.98 μm with 85:15 sample to 1.41 ± 0.69 μm with 50:50 sample. Conglutination was observed in microfibers of both 60:60 and 50:50 samples, although smaller-sized fibers were obtained. Increased PEO proportion could be the reason behind this phenomenon as it has a strong hygroscopicity which can absorb the moisture from the air and create a polymer flow instability due to the high cohesion of the polymeric solution.^31^

Fiber alignment is crucial in a drug delivery system as it can influence the release profile of active agents they carry. Aligned fibers exhibit more sustained release compared to random fibers.^32^ When the fibers are aligned, the release of any encapsulated substances, such as drugs or antibacterial agents, can occur in a more controlled and sustained manner. The alignment of fibers allows for a higher degree of interconnection creating channels or pathways through which the release can occur gradually over time.^32^ In contrast, random fibers may have a more disorganized structure, resulting in a less interconnected network. This can lead to faster and less controlled release kinetics compared to aligned fibers.^33^ Accordingly, 70:30 PP was selected for further Tri loading as the optimal blend since it displays a uniform fibrous structure, highly aligned fibers without the sign of a merged morphology, a smooth texture, and an appropriate level of production yield (%) (Figure 3A).

After the optimum PP ratio was determined as 70:30 (w/w), Tri was combined with the polymer blend as 5, 10, 20, 30, and 40% (weight of the polymer ratio). Corresponding calculated drug loading (%) and encapsulation efficiency (%) of pressure-spun TPP fibrous sutures and the loaded Tri amount per 1 mg fibrous suture sample are given in Table 2.

**Table 2:** Calculated Drug Loading (%), Encapsulation Efficiency (%), and Loaded Tri Amount (μg/mg) for Pressure-Spun Fibrous Suture Samples

sample	drug loading (%)	encapsulation efficiency (%)	loaded Tri amount (μg/mg)
5 TPP	4.9 ± 0.1	99.4 ± 2.5	49 ± 1
10 TPP	9.7 ± 0.1	97.5 ± 0.9	97 ± 1
20 TPP	19.3 ± 0.4	96.4 ± 2.1	193 ± 4
30 TPP	22.8 ± 0.3	76.1 ± 0.8	228 ± 3
40 TPP	28.5 ± 1.2	71.2 ± 3.1	285 ± 12

**Figure 4:**
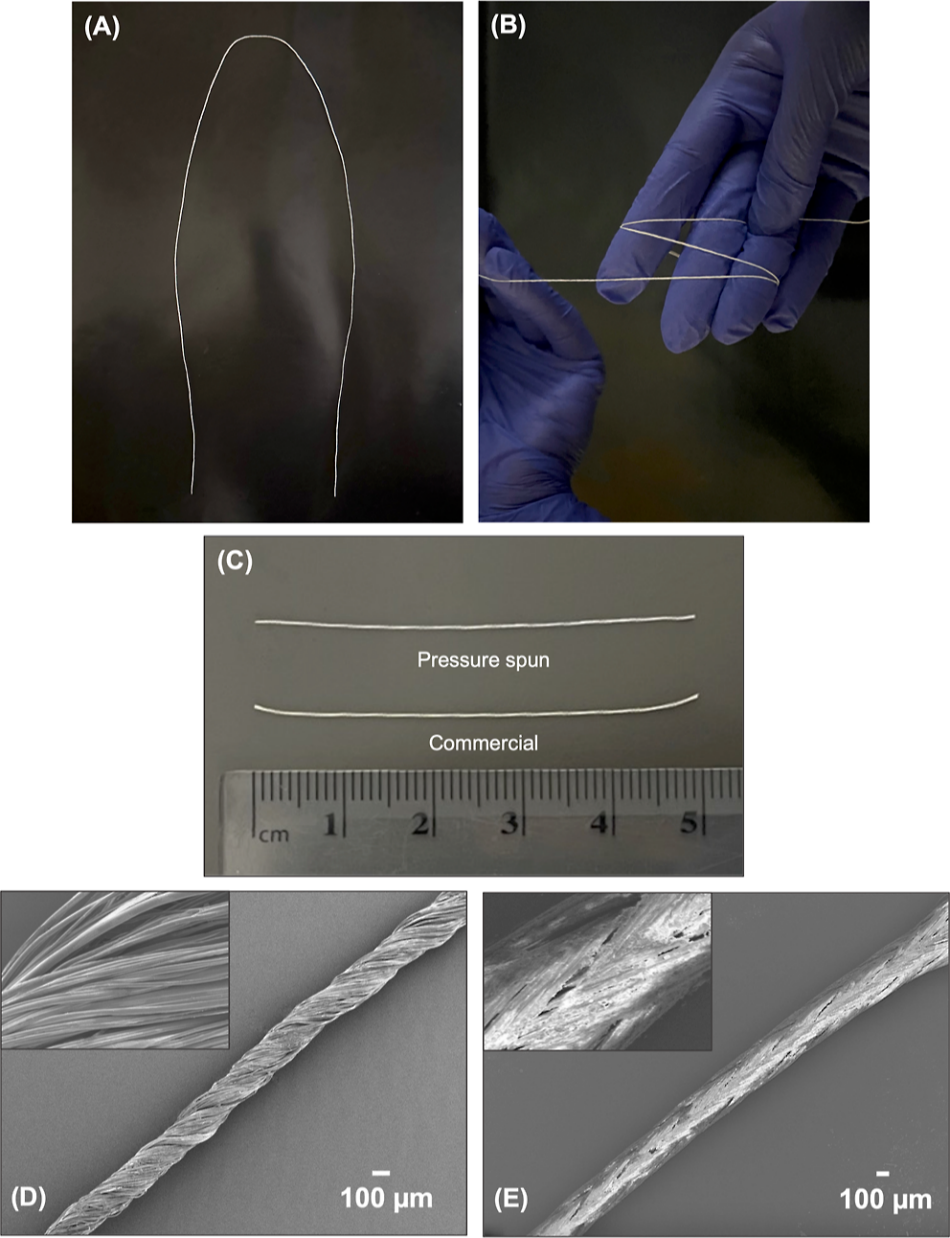
Macroscopic images of the pressure-spun 40 TPP fibrous suture (A), its handling (B), and comparison between the pressure-spun 40 TPP fibrous suture and commercial Vicryl Plus suture (C). SEM images showing pressure-spun 40 TPP fibrous suture (D) and SEM images of commercial Vicryl Plus suture (E).

Figure 4A displays the twisted pressure-spun TPP fibrous suture obtained. The pressure-spun TPP fibrous suture is characterized by its unique helical morphology, which provides increased flexibility and handling capabilities (Figure 4B). This makes them highly suitable for use as surgical sutures, as they can be easily manipulated and maneuvered during surgical procedures.^34^Figure 4C shows the manufactured 40 TPP fibrous suture and the commercial Vicryl Plus suture, exhibiting how the 40 TPP fibrous suture can macroscopically mimic the commercial Vicryl Plus suture. When analyzed microscopically, the SEM image of 40 TPP fibrous sutures displayed smaller-sized and highly aligned fibers even when twisted as a suture product (Figure 4D) compared to 70:30 PP (Figure 3B). The addition of a plasticizer, in this case, Tri, can add a free volume to the system and directly influence fiber diameter by interrupting contact between overlapping chains and reducing solution viscosity.^35^ Accordingly, adding Tri to the PP system introduced a free volume and lowered the solution viscosity (Table 1), and therefore further reduced the fiber diameter (2.62 ± 0.63 μm). Moreover, compared to braided commercial Vicryl Plus suture coated with Tri (Figure 4E), the pressure-spun twisted 40 TPP fibrous suture loaded with Tri exhibited a smoother surface (Figure 4D). It is reported that a smooth surgical suture surface causes less trauma, especially in sensitive tissues, efficiently lowering the friction coefficient, and improves suture movement in the tissue.^36^

### 3.2 In Vitro Antibacterial Assay

**Figure 5:**
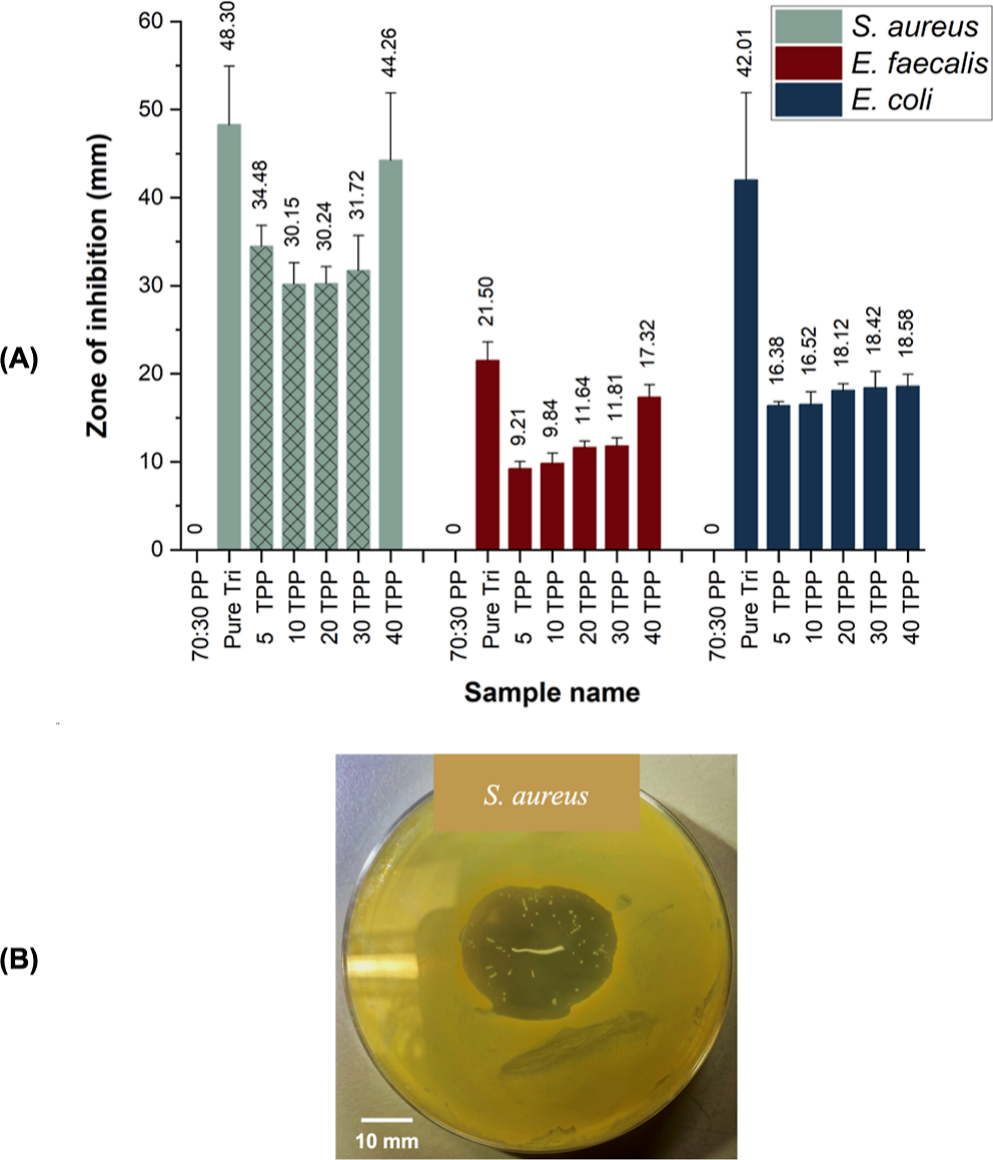
(A) Bar graph representation of the antibacterial activity of the Tri agent, pressure-spun 70:30 PP, and TPP fibrous sutures on *S. aureus*, *E. faecalis*, and *E. coli* strains. The hatched bars in the bar chart indicate the occurrence of resistant/regrowing bacteria observed within the tested Petri dishes. (B) A photograph of the TSA plate displaying bacterial colonies in the inhibition zone (white aggregated dots around the sample) after 5 TPP sutures incubated with *S. aureus*.

Effective Tri concentrations cause membrane damage through direct interaction and exert a bactericidal effect by blocking bacterial fatty acid biosynthesis.^37^ The antibacterial activity of the Tri agent (positive control), 70:30 PP (negative control), and pressure-spun TPP fibrous sutures was further evaluated using an in vitro agar diffusion assay against *S. aureus*, *E. faecalis*, and *E. coli* strains. Samples were cultured for 24 h at 37 °C, and antibacterial activity was determined by measuring the diameter of the inhibition zone around the suture samples (Figure 5A). Resistant bacteria were observed in Petri dishes containing 5, 10, 20, and 30 TPP fibrous sutures tested with *S. aureus* (Figure 5B). Though a clear radius can be observed, there are a number of colonies growing within the clear zone. This suggests that several colonies are resistant to Tri or there is regrowth of the bacteria as the concentration of Tri in the fibers is not strong enough. However, increased Tri concentration presented a clear inhibition zone with 40 TPP samples, indicating that the minimum Tri concentration loaded into fibrous sutures should be greater than 228 ± 3 μg/mg to combat with *S. aureus* microbe. All samples produced (5–40 TPP) showed inhibitory activity that increases with the concentration of the Tri loaded into fibrous sutures against both *E. faecalis* and *E. coli* strains. From Figure 5A, it can also be seen that the 40 TPP sample displayed similar antibacterial activity toward Gram-positive bacteria when compared to pure Tri, indicating the pressure-spun fibrous sutures are just as effective as the agent alone. The results presented here show 40 TPP fibrous sutures as a suitable alternative for the targeted treatment of SSIs, therefore in turn reducing the human consumption of antibiotics. Additionally, since the 40 TPP sample exhibited activity against all strains tested, it was taken as the model pressure-spun antibacterial fibrous suture for all remaining characterization tests.

**Figure 6:**
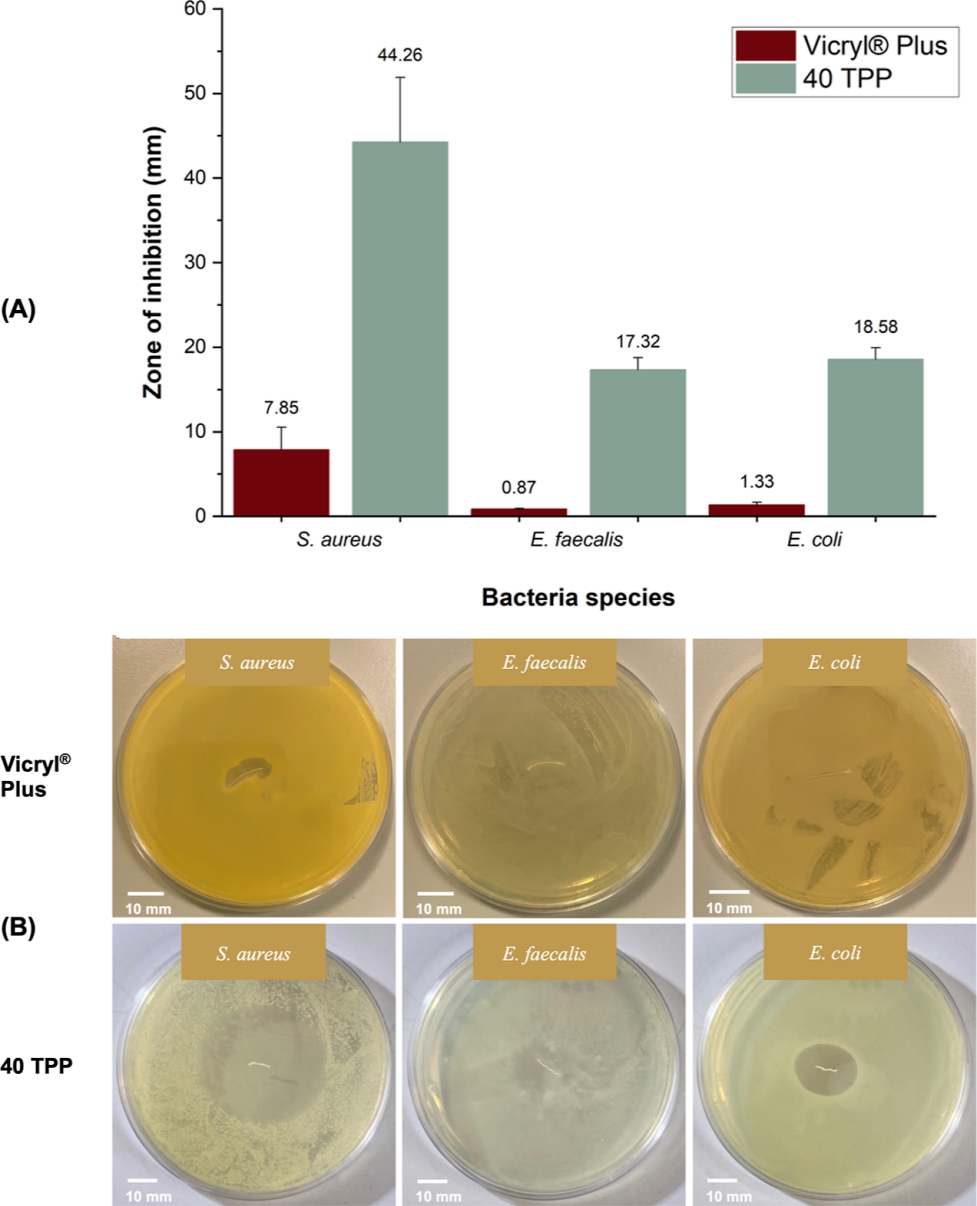
Bar graph comparison of the antibacterial activity of a commercial Vicryl Plus suture and pressure-spun 40 TPP fibrous suture (A). Photographs of the Petri dishes display inhibition zones of commercial Vicryl Plus suture and pressure-spun 40 TPP fibrous suture against *S. aureus*, *E. faecalis*, and *E. coli* strains (B).

Vicryl Plus has been reported to have a 472 μg/m Tri agent coating.^9^ In an in vitro antibacterial test, only 10 mm of the sample was used, which is estimated to have Tri coating up to 4.72 μg. The 40 TPP sample used in the test is again 10 mm and had a Tri loading of 71.25 ± 75 μg. As expected, 40 TPP samples loaded with a higher amount of Tri displayed a higher antibacterial activity compared to Vicryl Plus for all strains tested (Figure 6).

### 3.3 FTIR Spectral Analysis

**Figure 7:**
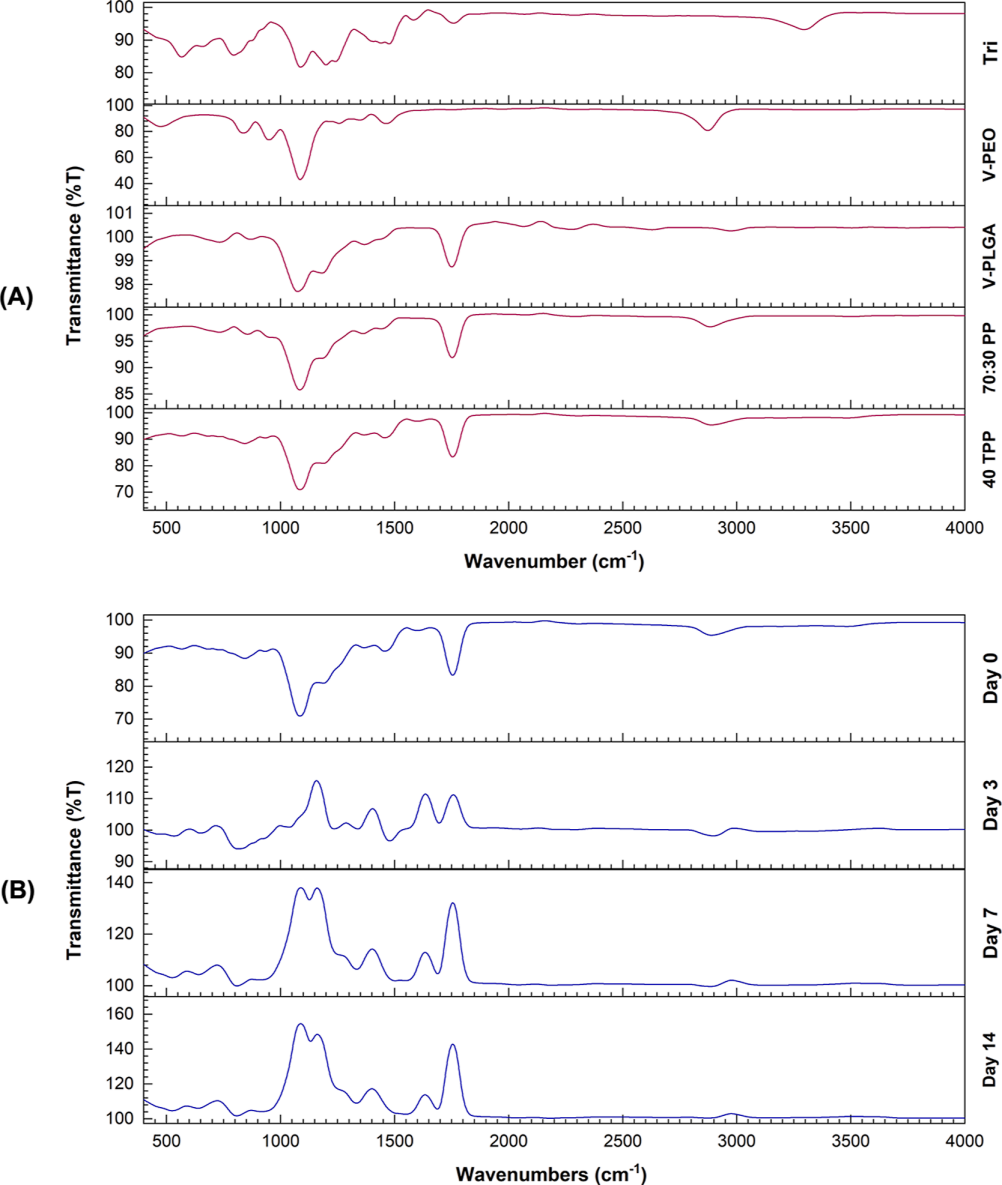
FTIR absorbance spectra for pressure-spun fibrous suture samples before (A) and after (B) biodegradation analysis in an aqueous environment (PBS, pH 7.4) for 14 days.

FTIR analysis was carried out to examine the incorporation of Tri agent in TPP fibrous surgical sutures, evaluate the possibility of a chemical interaction between polymers and the agent, and obtain and compare the spectra of Tri agent, V-PEO, V-PLGA, 70:30 PP, and 40 TPP fibrous surgical sutures. As shown in Figure 7A, PP exhibited absorption peaks at 2999 and 2884 cm^–1^ (C–H stretch), 1689 cm^–1^ (C=O stretch), regions from 1452 to 1085 cm^–1^ (C–O stretch), and region between 952 and 841 cm^–1^ (rocking vibrations of CH_2_ groups).^38^ Characteristic identification peaks of Tri were detected at 1754 (C=O stretch), 1596 and 1473 (C–H stretch), 913 (C–H bend), and C–Cl stretching peaks were observed at 800–592 cm^–1^ (C–Cl stretch).^39^ No chemical interactions (new peaks or shifting) were observed, indicating that pressurized gyration did not affect the functional groups of polymers and the Tri agent. Tri agent was physically bound to 70:30 PP and can be well released from the fibrous suture with an initial burst release that provides a high initial antibacterial activity.^11^

Dissolution of polymers and release of Tri were also confirmed by FTIR analysis after the degradation of a pressure-spun 40 TPP fibrous suture in an aqueous environment (PBS, pH 7.4) for 14 days (Figure 7B). Characteristic peaks of Tri agent in 40 TPP fibrous sutures were no longer observed in degraded samples, characteristic signals of PEO at 2884, 1085 cm^–1^, and between 952 and 841 cm^–1^ decreased with the dissolution of PEO, and final spectra overlapped with V-PLGA spectrum at the end of 14 day period.

### 3.4 In Vitro Degradation and Surface Wettability

**Figure 8:**
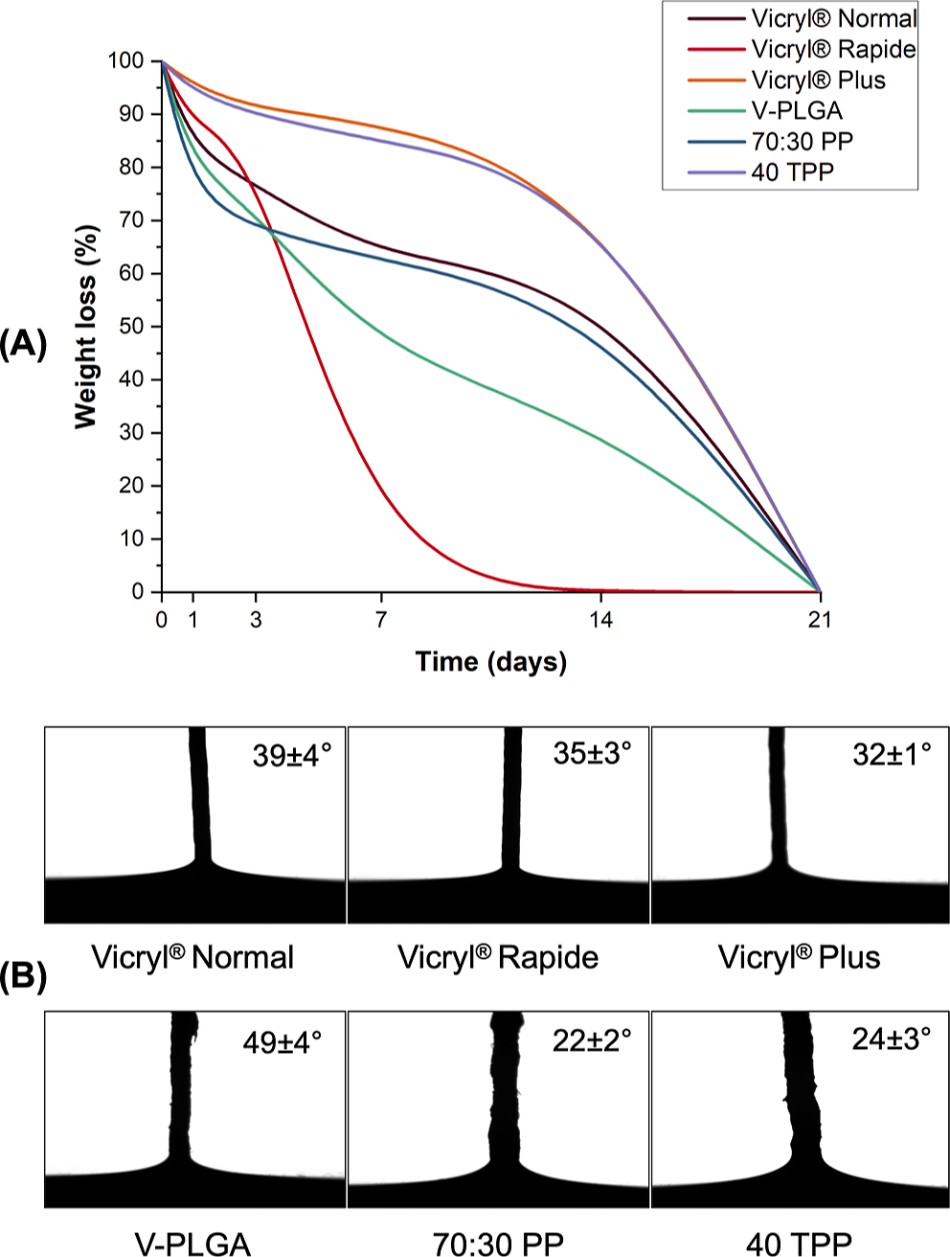
(A) Weight loss (%) of pressure-spun V-PLGA, 70:30 PP, 40 TPP fibrous surgical sutures, and commercial Vicryl Normal, Vicryl Rapide, and Vicryl Plus sutures post incubation in PBS (pH 7.4) at 37 °C for 21 days. (B) WCA values of commercial Vicryl Normal, Vicryl Rapide, and Vicryl Plus sutures along with pressure-spun V-PLGA, 70:30 PP, and 40 TPP fibrous surgical sutures.

Weight loss (%) of V-PLGA, 70:30 PP, and 40 TPP fibrous surgical sutures and commercial Vicryl Normal, Vicryl Rapide, and Vicryl Plus sutures was analyzed in an aqueous medium (PBS, pH 7.4) at 37 °C for 21 days. As can be observed from Figure 8A, a major weight loss occurred within 7 days of the degradation period for samples not having Tri agent. On day 7, the remaining fibrous suture weights were 44.39, 62.62, and 84.97% for V-PLGA, 70:30 PP, and 40 TPP and 62.38, 1.86, and 88.46% for Vicryl Normal, Vicryl Rapide, and Vicryl Plus, respectively. Between 7 and 14 days, no significant changes were observed in the residual weights of the samples, except Vicryl Rapide which degraded completely. Vicryl Rapide is formed by irradiating Vicryl Normal and is considered to cause chain scission in PGA polymers, reducing its molecular weight and accelerating its degradation.^40^ Therefore, it is believed that it completely degraded before other sutures were tested. For the rest, complete degradation was observed between 14 and 21 days. Vicryl Plus and pressure-spun 40 TPP fibrous sutures exhibited a similar weight loss profile over the degradation period, confirming that their degradation behaviors in an aqueous medium are comparable.

Surface wettability (hydrophobicity/hydrophilicity when in contact with water) is an important property that can affect the biological response of an implanted material.^41^ The surface wettability of pressure-spun V-PLGA, 70:30 PP, 40 TPP fibrous surgical sutures, and commercial Vicryl Normal, Vicryl Rapide, and Vicryl Plus sutures was tested by measuring the water contact angle (WCA). Implanted materials with hydrophilic surfaces improve protein adsorption, exhibiting improved cell adhesion and proliferation.^42^ All samples tested in this study were found to be hydrophilic as their value of the angles was lower than 90°.^43^ According to the results, V-PLGA fibrous suture (WCA of 49 ± 4°) exhibited a lower hydrophilic nature compared to commercial Vicryl Normal (WCA of 39 ± 4°). This hydrophilicity of V-PLGA increased by more than 50% when PLGA blended with PEO in 70:30 PP fibrous surgical sutures (WCA of 22 ± 2°). Moreover, this blend again displayed advanced hydrophilicity compared to that of the Vicryl Rapide suture. Increased hydrophilicity also increases water uptake, resulting in a higher degradation rate.^44^ Although 70:30 PP showed a higher degree of hydrophilicity, it did undergo a slower weight loss compared with Vicryl Rapide suture samples (Figure 8B). The reduced molecular weight of Vicryl Rapide during the irradiation of Vicryl Normal can be the reason behind this phenomenon.^40^ 40 TPP sample exhibited similar WCA to 70:30 PP, but a higher hydrophilic nature than the Vicryl Plus.

### 3.5 Thermal Properties

**Figure 9:**
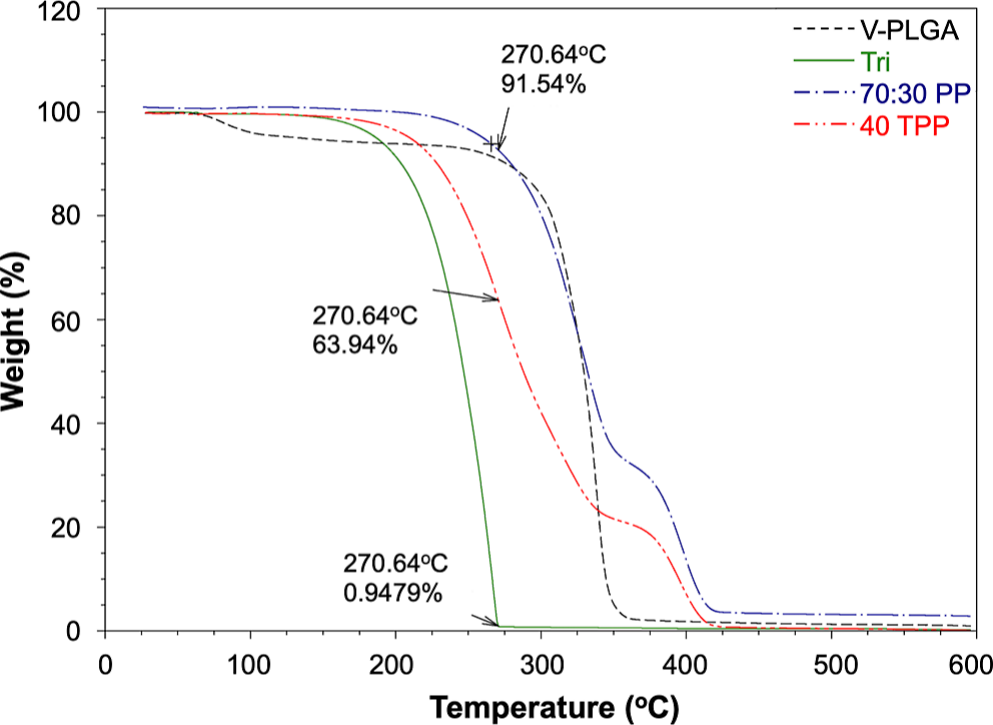
(A) TGA thermograms of the Tri agent, V-PLGA, 70:30 PP, and 40 TPP fibrous surgical sutures.

TGA was used to assess the thermal properties of the Tri agent, pressure-spun V-PLGA, and 70:30 PP fibrous surgical sutures and to investigate the combination of Tri within the 40 TPP fibrous surgical sutures. The overlay of TGA thermograms of samples is shown in Figure 9. All samples were found suitable for use as suture products in surgical operations as they did not exhibit any decomposition at both ambient and body temperatures (25 and 37 °C).^38^ V-PLGA fibrous surgical suture started its weight loss at ∼250 °C and completed the thermal decomposition with constant residual mass when the temperature reached ∼360 °C, which was similar to the results obtained by Almajhdi et al.^45^ The pure Tri agent showed a simple one major weight loss profile with a single transition temperature and finished its decomposition after 270 °C. The weight loss percentages of 70:30 PP and 40 TPP at around 270 °C were calculated as 8.46 and 36.06%, respectively, suggesting that approximately 27.6% of Tri was successfully incorporated into the fibrous surgical sutures, which is almost equal to 69% of the initially added weight of Tri agent. This result also indicates that 1 mg (40 mm) of a 40 TPP fibrous surgical suture has a Tri loading of 0.276 mg, confirming the calculated amount presented in Table 2. The DSC results are given in the Supporting Information (Figure S1).

### 3.6 Mechanical Properties

**Figure 10:**
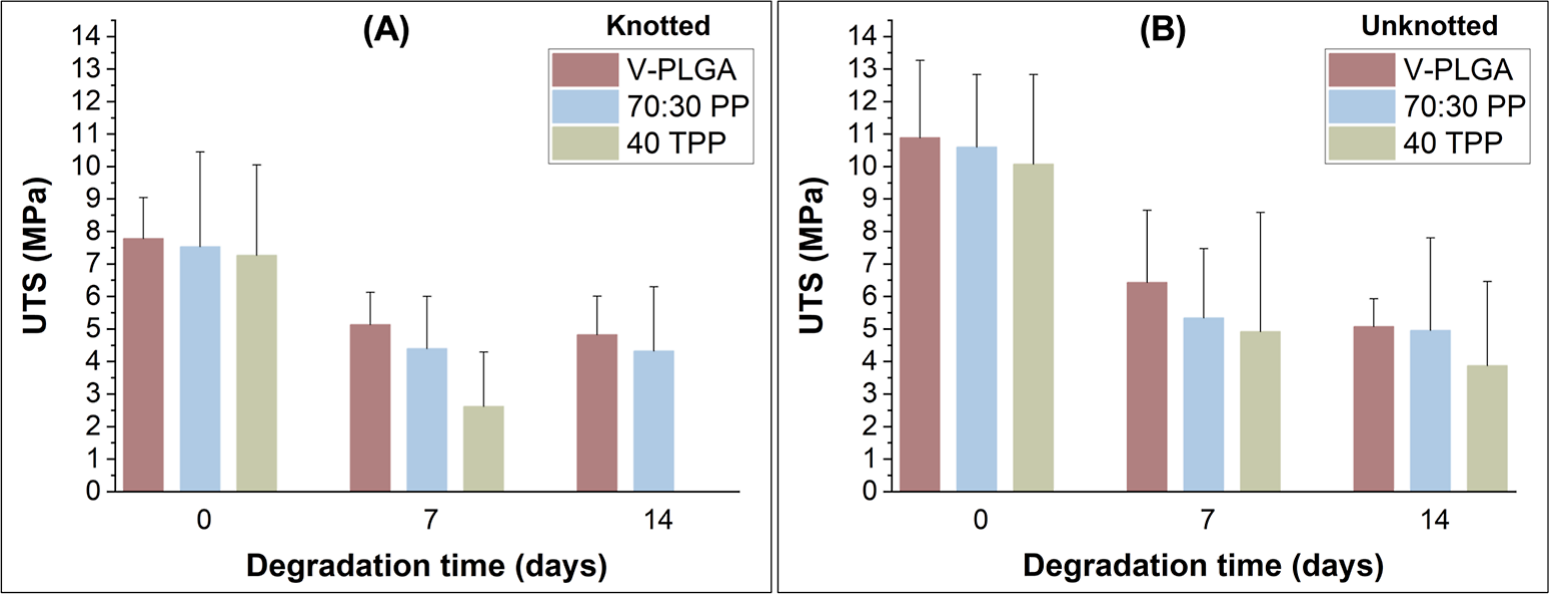
UTS (MPa) values of unknotted (A) and knotted (B) pressure-spun V-PLGA, 70:30 PP, and 40 TPP fibrous surgical sutures before, during, and after 14 days of degradation.

Tensile testing was performed on pressure-spun V-PLGA, 70:30 PP, and 40 TPP fibrous surgical sutures to determine the UTS in both unknotted and square-knotted forms. The UTS of 7 and 14 days degraded fibrous surgical sutures and was also investigated. The results for all conditions are given in Figure 10. Before any degradation, all samples exhibited similar UTS values in their unknotted forms. The existence of a knot is expected to reduce the mechanical strength of the suture fibers as the knot is a region with high-stress concentration rather than a point along the fibrous structure.^46^ With knotting, a lower UTS of V-PLGA (7.77 ± 1.26 MPa) was measured compared to its unknotted form (10.88 ± 2.38 MPa). The same trend was also observed when PLGA was blended with PEO in 70:30 PP fibrous surgical suture (from 10.59 ± 2.24 to 7.53 ± 2.92 MPa) and for 40 TPP samples, from 10.07 ± 2.76 to 7.26 ± 2.78 MPa.

After 7 days of degradation in the aqueous medium, UTS values of all unknotted and knotted fibrous surgical sutures were significantly lower than the ones in their dry state as they began to lose their strength due to hydrolysis.^47^ The UTS value for all samples almost halved in 7 days in both forms. Release of Tri from 40 TPP fibrous sutures in the aqueous medium during the degradation testing reduced the tensile strength of the suture in both unknotted and knotted forms. Even though its unknotted form had a UTS of 3.86 ± 2.59 MPa, the knotted 40 TPP degraded to the point where tensile testing was extremely difficult after 14 days of degradation, as the suture would disintegrate when manipulated and therefore could not be tested.

Depending on the surgical area of application, the required original tensile strength must be guaranteed so that the suture material does not break and support the wound.^48^ The remaining approximate original strength of Vicryl Plus in unknotted form is reported to be 75% at 14 days after implantation,^9^ while the pressure-spun 40 TPP fibrous suture retained 38% of its original strength on that day. On the other hand, unknotted Vicryl Rapide almost completely loses its original breaking strength in 14 days.^49^ Overall, all pressure-spun fibrous sutures tested had adequate tensile strength.^50^ Accordingly, the 40 TPP fibrous suture is expected to combine the faster absorption property of Vicryl Rapide and the antibacterial properties of Vicryl Plus in a single pressure-spun fibrous suture.

### 3.7 In Vitro Drug Release and Cell Viability

**Figure 11:**
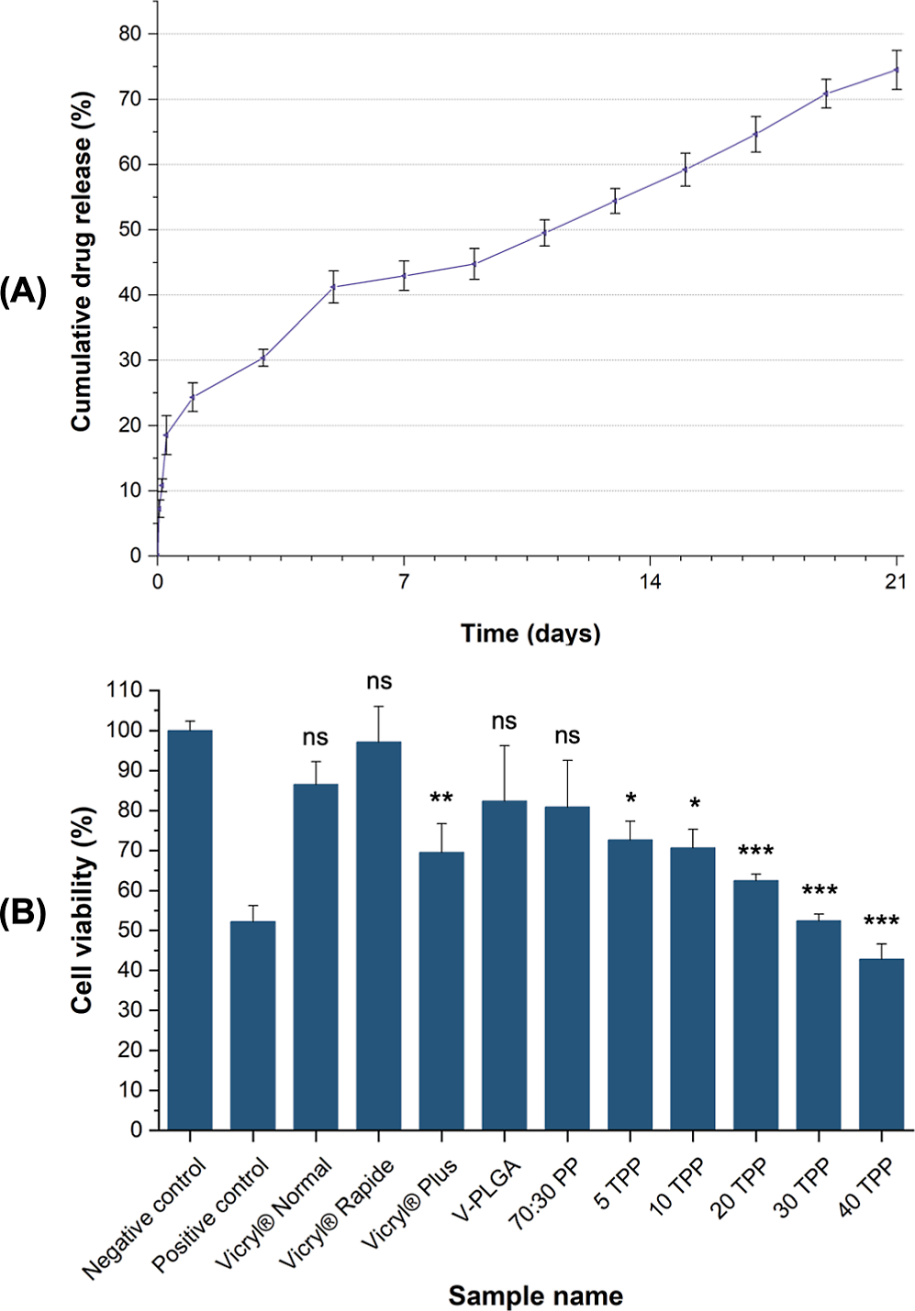
(A) Cumulative drug release (%) profile of pressure-spun 40 TPP fibrous suture after 21 days of in vitro release in PBS (pH 7.4) at 37 °C. (B) Cell viability (%) of pressure-spun TPP fibrous sutures (5–40) and commercial Vicryl Normal, Vicryl Rapide, and Vicryl Plus sutures for 24 h of incubation. Each value represents mean ± SD; *n* = 3, where, *p < 0.005; **p < 0.001; ***p < 0.0001; ns = not significant as compared to the negative control.

Figure 11A displays the cumulative release (%) of Tri agent from 40 TPP fibrous sutures into the PBS (pH 7.4) release environment for 21 days. The pressure-spun fibrous suture exhibited an initial burst release of Tri within 24 h (24.35%, 17.35 ± 0.73 μg), followed by a sustained release for the remainder until the fibrous suture completely degraded in 21 days (Figure 8A). The final percentage of Tri release from 40 TPP fibrous sutures was 74.52% (53.10 ± 2.24 μg). A release profile with an initial burst phase with a subsequent sustained release is desirable for combating bacteria since the initial release of dose-dependent agents like Tri can prevent infection and provide long-term antibacterial activity in the later phase.^51,52^

The choice of specific materials and an antibacterial agent in this study was based on commercial Vicryl products. However, it is important to acknowledge that the selection of these components can have a significant effect on in vitro–in vivo correlations (IVIVC). IVIVC is a predictive mathematical model that describes the relationship between an in vitro property of a dosage form and the corresponding in vivo response.^53^ It plays a pivotal role in predicting the in vivo performance of a drug product based on its in vitro drug release profile, optimizing formulations to achieve desired in vivo release profiles, setting dissolution limits, and reducing the need for extensive bioequivalence studies during product development.^54^

By altering the components used in the pressure-spun fibrous surgical sutures, the release behavior can be tailored to more closely align with the desired in vivo performance. For instance, selecting different biodegradable materials with varying degradation rates can influence the sustained release profile of the antibacterial agent from the pressure-spun fibrous suture. This modification enables better mimicry of the degradation kinetics observed in vivo, leading to improved IVIVC. Additionally, choosing antibacterial agents with specific characteristics, such as controlled release properties or different mechanisms of action, can enhance the suture’s antibacterial efficacy in vivo. Careful selection of the antibacterial agent allows for alignment of the release behavior with the needs of the surgical site, ensuring long-lasting and effective antibacterial activity.

It is anticipated that future studies involving altered materials and antibacterial agents will provide valuable insights into IVIVC. These studies should include in vivo evaluations to assess the performance of the modified fibrous suture in relevant animal models or human subjects, considering factors such as the host’s immune response, tissue integration, wound healing, and overall clinical outcomes.

Figure 11B exhibits colorimetric MTT assay results for the quantitative assessment of cytotoxicity on L929 cells. Results indicate that the non-Tri-containing samples have no toxic effects on mammalian cells in vitro. Cell viability (%) of pressure-spun TPP fibrous sutures shows a decreasing trend as the Tri content increases compared to the negative control. This finding aligns with previous studies in the literature, which have shown that the incorporation of antibacterial agents, such as Tri, can have an impact on cell viability.^55–57^ The cytotoxicity of Tri has been reported with respect to various cell types, and its effects can be dose-dependent.^58^ It is worth noting that in this study the cell viability remains at 70% and above, up to the 20 TPP sample. However, as the Tri content further increases, it is observed that the cell viability decreases. These results suggest that the cytotoxic effects of Tri-loaded pressure-spun TPP fibrous sutures should be carefully considered when determining their suitability for specific applications. The materials and formulations used in this study were chosen as a starting point to explore the feasibility and potential of pressure-spun TPP fibrous sutures with Tri content. The results and insights gained from this work will serve as valuable guidance for the subsequent selection and development of more appropriate materials and formulations. By considering alternative materials and adjusting the Tri content, it will be possible to conduct more in-depth evaluations and validations of the suture’s biocompatibility and cytotoxic effects. By building upon these initial findings and incorporating advancements in material selection and formulation, further investigations in an in vivo animal model will contribute to the refinement and optimization of the pressure-spun fibrous suture design, ultimately leading to the development of safer and more effective sutures for potential clinical applications. Finally, well-designed randomized controlled trials should be undertaken to rigorously evaluate the long-term safety and efficacy of the sutures, including wound healing studies and assessments of the pressure-spun fibrous suture’s antibacterial properties in SSIs.

## 4 Conclusions

The research demonstrates a novel engineering approach to mass produce an absorbable fibrous surgical suture with antibacterial properties, based on an innovative manufacturing design. Pressurized gyration was used for the first time in the production of surgical sutures by V-PLGA, V-PEO, physically blended PP and the antibacterial Tri agent-loaded PP. 285 ± 12 μg/mg Tri-loaded sample was chosen as a model antibacterial fibrous suture to compare with commercially available and FDA- and NICE-approved antibacterial Vicryl Plus suture after in vitro antibacterial assay. Model pressure-spun antibacterial fibrous sutures exhibited a similar weight loss profile but a higher hydrophilic nature compared to Vicryl Plus sutures. It also exhibited a sustained release profile over a 21 day period starting with an initial burst release. Additionally, all pressure-spun fibrous sutures did not show any decomposition at both ambient and body temperatures (25 and 37 °C), and mechanical properties were suitable to ensure proper wound healing. According to all characterization test results, the pressure-spun antibacterial fibrous suture explored in this work is expected to combine the faster absorption property of Vicryl Rapide and the antibacterial properties of Vicryl Plus in a single suture. On the other hand, our ongoing exploration of biodegradable materials, antibacterial agents, and safer solvents, as well as the conduct of in vivo evaluations to assess mechanical properties, drug release (with the establishment of IVIVC), biodegradability, wound healing capabilities, biocompatibility, bacteria killing efficacy, and toxicity, will enhance our understanding of the fibrous sutures’ performance in real-world settings and contribute to the advancement of surgical interventions. Overall, with its simplicity and ability to manufacture sutures from almost any polymer-drug blend, the pressurized gyration method has the potential to combat SSIs and emerging antibacterial resistance when used for the development of antibacterial surgical sutures with localized delivery ability.

## Supporting Information Available

Additional characterization results (PDF)

## References

[ref1] UK Health Security Agency (UKHSA). Protocol for the Surveillance of Surgical Site Infection, 2022. https://assets.publishing.service.gov.uk/government/uploads/system/uploads/attachment_data/file/1048707/Protocol_for_the_Surveillance_of_Surgical_Site_Infection.pdf (accessed July 28, 2023).

[ref2] GIRFT SSI National Survey, 2019. https://gettingitrightfirsttime.co.uk/wp-content/uploads/2017/08/SSI-Report-GIRFT-APRIL19e-FINAL.pdf (accessed July 28, 2023).

[ref3] UK Department of Health and Social Care. UK’s five-year national action plan, Tackling antimicrobial resistance 2019–2024, 2019. https://www.gov.uk/government/publications/uk-5-year-action-plan-for-antimicrobial-resistance-2019-to-2024 (accessed July 28, 2023).

[ref4] Public Health England. PHE Strategy 2020–25, 2019. https://www.gov.uk/government/publications/phe-strategy-2020-to-2025 (accessed July 28, 2023).

[ref5] Scott Taylor M.; Shalaby S. W. Chapter II. 5.15—Sutures. In Biomaterials Science, 3rd ed.; Ratner B. D., Hoffman A. S., Schoen F. J., Lemons J. E., Eds.; Academic Press, 2013; pp 1010–1024.

[ref6] Duarah R.; Singh Y. P.; Gupta P.; Mandal B. B.; Karak N. Smart self-tightening surgical suture from a tough bio-based hyperbranched polyurethane/reduced carbon dot nanocomposite. Biomed. Mater. 2018, 13, 045004 10.1088/1748-605X/aab93c. 29570096

[ref7] Vieira D.; Angel S. N.; Honjol Y.; Masse M.; Gruenheid S.; Harvey E. J.; Merle G. Engineering Surgical Stitches to Prevent Bacterial Infection. Sci. Rep. 2022, 12 (1), 834 10.1038/s41598-022-04925-5. 35039588 PMC8764053

[ref8] Ahmed I.; Boulton A. J.; Rizvi S.; Carlos W.; Dickenson E.; Smith N. A.; Reed M. The Use of Triclosan-Coated Sutures to Prevent Surgical Site Infections: A Systematic Review and Meta-Analysis of the Literature. BMJ Open 2019, 9 (9), e029727 10.1136/bmjopen-2019-029727. PMC6731927 31481559

[ref9] National Institute for Health and Care Excellence (NICE). Plus Sutures for preventing surgical site infection, 2021. https://www.nice.org.uk/guidance/mtg59/resources/plus-sutures-for-preventing-surgical-site-infection-pdf-64372124642245#:~:text=Plus%20Sutures%20is%20innovative%20because,for%20at%20least%207%20dayshttps://www.nice.org.uk/guidance/mtg59/resources/plus-sutures-for-preventing-surgical-site-infection-pdf-64372124642245#:~:text=Plus%20Sutures%20is%20innovative%20because,for%20at%20least%207%20days (accessed July 28, 2023).

[ref10] Altun E.; Aydogdu M. O.; Crabbe-Mann M.; Ahmed J.; Brako F.; Karademir B.; Aksu B.; Sennaroglu M.; Eroglu M. S.; Ren G.; Gunduz O.; Edirisinghe M. Co-Culture of Keratinocyte-Staphylococcus aureus on Cu-Ag-Zn/CuO and Cu-Ag-W Nanoparticle Loaded Bacterial Cellulose:PMMA Bandages. Macromol. Mater. Eng. 2019, 304 (1), 1800537 10.1002/mame.201800537.

[ref11] Altun E.; Yuca E.; Ekren N.; Kalaskar D. M.; Ficai D.; Dolete G.; Ficai A.; Gunduz O. Kinetic Release Studies of Antibiotic Patches for Local Transdermal Delivery. Pharmaceutics 2021, 13 (5), 613 10.3390/pharmaceutics13050613. 33922739 PMC8145298

[ref12] Brako F.; Thorogate R.; Mahalingam S.; Raimi-Abraham B.; Craig D. Q. M.; Edirisinghe M. Mucoadhesion of Progesterone-Loaded Drug Delivery Nanofiber Constructs. ACS Appl. Mater. Interfaces 2018, 10 (16), 13381–13389. 10.1021/acsami.8b03329. 29595052

[ref13] Basnett P.; Matharu R. K.; Taylor C. S.; Illangakoon U.; Dawson J. I.; Kanczler J. M.; Behbehani M.; Humphrey E.; Majid Q.; Lukasiewicz B.; Nigmatullin R.; Heseltine P.; Oreffo R. O. C.; Haycock J. W.; Terracciano C.; Harding S. E.; Edirisinghe M.; Roy I. Harnessing Polyhydroxyalkanoates and Pressurized Gyration for Hard and Soft Tissue Engineering. ACS Appl. Mater. Interfaces 2021, 13 (28), 32624–32639. 10.1021/acsami.0c19689. 34228435

[ref14] Ahmed J.; Matharu R. K.; Shams T.; Illangakoon U. E.; Edirisinghe M. A Comparison of Electric-Field-Driven and Pressure-Driven Fiber Generation Methods for Drug Delivery. Macromol. Mater. Eng. 2018, 303 (5), 1700577 10.1002/mame.201700577.

[ref15] Arora A.; Aggarwal G.; Chander J.; Maman P.; Nagpal M. Drug Eluting Sutures: A Recent Update. J. Appl. Pharm. Sci. 2019, 9 (7), 111–123. 10.7324/JAPS.2019.90716.

[ref16] Padmakumar S.; Joseph J.; Neppalli M. H.; Mathew S. E.; Nair S. V.; Shankarappa S. A.; Menon D. Electrospun Polymeric Core-Sheath Yarns as Drug Eluting Surgical Sutures. ACS Appl. Mater. Interfaces 2016, 8 (11), 6925–6934. 10.1021/acsami.6b00874. 26936629

[ref17] Mahalingam S.; Edirisinghe M. Forming of Polymer Nanofibers by a Pressurised Gyration Process. Macromol. Rapid Commun. 2013, 34 (14), 1134–1139. 10.1002/marc.201300339. 23749758

[ref18] Heseltine P. L.; Ahmed J.; Edirisinghe M. Developments in Pressurized Gyration for the Mass Production of Polymeric Fibers. Macromol. Mater. Eng. 2018, 303 (9), 1800218 10.1002/mame.201800218.

[ref19] Sun X.; Xu C.; Wu G.; Ye Q.; Wang C. Poly(Lactic-Co-Glycolic Acid): Applications and Future Prospects for Periodontal Tissue Regeneration. Polymers 2017, 9 (12), 189 10.3390/polym9060189. 30970881 PMC6432161

[ref20] Bae S.; DiBalsi M. J.; Meilinger N.; Zhang C.; Beal E.; Korneva G.; Brown R. O.; Kornev K. G.; Lee J. S. Heparin-Eluting Electrospun Nanofiber Yarns for Antithrombotic Vascular Sutures. ACS Appl. Mater. Interfaces 2018, 10 (10), 8426–8435. 10.1021/acsami.7b14888. 29461035

[ref21] Ostuni E.; Chapman R. G.; Holmlin R. E.; Takayama S.; Whitesides G. M. A Survey of Structure-Property Relationships of Surfaces That Resist the Adsorption of Protein. Langmuir 2001, 17 (18), 5605–5620. 10.1021/la010384m.

[ref22] Makadia H. K.; Siegel S. J. Poly Lactic-Co-Glycolic Acid (PLGA) as Biodegradable Controlled Drug Delivery Carrier. Polymers 2011, 3 (3), 1377–1397. 10.3390/polym3031377. 22577513 PMC3347861

[ref23] Parikh K. S.; Omiadze R.; Josyula A.; Shi R.; Anders N. M.; He P.; Yazdi Y.; McDonnell P. J.; Ensign L. M.; Hanes J. Ultra-thin, high strength, antibiotic-eluting sutures for prevention of ophthalmic infection. Bioeng. Transl. Med. 2021, 6, e10204 10.1002/btm2.10204. 34027091 PMC8126818

[ref24] Alenezi H.; Cam M. E.; Edirisinghe M. Experimental and Theoretical Investigation of the Fluid Behavior during Polymeric Fiber Formation with and without Pressure. Appl. Phys. Rev. 2019, 6 (4), 041401 10.1063/1.5110965.

[ref25] Altun E.; Ahmed J.; Onur Aydogdu M.; Harker A.; Edirisinghe M. The effect of solvent and pressure on polycaprolactone solutions for particle and fibre formation. Eur. Polym. J. 2022, 173, 111300 10.1016/j.eurpolymj.2022.111300.

[ref26] Altun E.; Aydogdu M. O.; Koc F.; Crabbe-Mann M.; Brako F.; Kaur-Matharu R.; Ozen G.; Kuruca S. E.; Edirisinghe U.; Gunduz O.; Edirisinghe M. Novel Making of Bacterial Cellulose Blended Polymeric Fiber Bandages. Macromol. Mater. Eng. 2018, 303 (3), 1700607 10.1002/mame.201700607.

[ref27] https://www.jnjmedtech.com/en-GB/product/coated-vicryl-plus-antibacterial-polyglactin-910-suture (accessed July 28, 2023).

[ref28] Hansen D.; Bomholt N.; Jeppesen J. C.; Simonsen A. C. Contact angle goniometry on single micron-scale fibers for composites. Appl. Surf. Sci. 2017, 392, 181–188. 10.1016/j.apsusc.2016.09.018.

[ref29] Hong X.; Edirisinghe M.; Mahalingam S. Beads, beaded-fibres and fibres: Tailoring the morphology of poly (caprolactone) using pressurised gyration. Mater. Sci. Eng., C 2016, 69, 1373–1382. 10.1016/j.msec.2016.07.071. 27612839

[ref30] Evrova O.; Hosseini V.; Milleret V.; Palazzolo G.; Zenobi-Wong M.; Sulser T.; Buschmann J.; Eberli D. Hybrid Randomly Electrospun Poly(Lactic- *Co* -Glycolic Acid):Poly(Ethylene Oxide) (PLGA:PEO) Fibrous Scaffolds Enhancing Myoblast Differentiation and Alignment. ACS Appl. Mater. Interfaces 2016, 8 (46), 31574–31586. 10.1021/acsami.6b11291. 27726370

[ref31] Juarez-Enriquez E.; Olivas G. I.; Zamudio-Flores P. B.; Ortega-Rivas E.; Perez-Vega S.; Sepulveda D. R. Effect of Water Content on the Flowability of Hygroscopic Powders. J. Food Eng. 2017, 205, 12–17. 10.1016/j.jfoodeng.2017.02.024.

[ref32] Eslamian M.; Khorrami M.; Yi N.; Majd S.; Abidian M. R. Electrospinning of Highly Aligned Fibers for Drug Delivery Applications. J. Mater. Chem. B 2019, 7 (2), 224–232. 10.1039/C8TB01258J. 31372224 PMC6675471

[ref33] Luraghi A.; Peri F.; Moroni L. Electrospinning for drug delivery applications: A review. J. Controlled Release 2021, 334, 463–484. 10.1016/j.jconrel.2021.03.033. 33781809

[ref34] Byrne M.; Aly A. The Surgical Suture. Aesthetic Surg. J. 2019, 39, S67–S72. 10.1093/asj/sjz036. 30869751

[ref35] Zamani M.; Morshed M.; Varshosaz J.; Jannesari M. Controlled Release of Metronidazole Benzoate from Poly ε-Caprolactone Electrospun Nanofibers for Periodontal Diseases. Eur. J. Pharm. Biopharm. 2010, 75 (2), 179–185. 10.1016/j.ejpb.2010.02.002. 20144711

[ref36] Zhang G.; Ren T.; Zeng X.; Van Der Heide E. Influence of Surgical Suture Properties on the Tribological Interactions with Artificial Skin by a Capstan Experiment Approach. Friction 2017, 5 (1), 87–98. 10.1007/s40544-017-0140-3.

[ref37] Nudera W. J.; Fayad M. I.; Johnson B. R.; Zhu M.; Wenckus C. S.; BeGole E. A.; Wu C. D. Antimicrobial Effect of Triclosan and Triclosan with Gantrez on Five Common Endodontic Pathogens. J. Endod. 2007, 33 (10), 1239–1242. 10.1016/j.joen.2007.06.009. 17889698

[ref38] Eroglu I.; Gultekinoglu M.; Bayram C.; Erikci A.; Ciftci S. Y.; Ayse Aksoy E.; Ulubayram K. Gel Network Comprising UV Crosslinked PLGA-b-PEG-MA Nanoparticles for Ibuprofen Topical Delivery. Pharm. Dev. Technol. 2019, 24 (9), 1144–1154. 10.1080/10837450.2019.1643880. 31298072

[ref39] Özişik H.; Bayari S. H.; Sağlam S.; Angelopoulos A.; Fildisis T. Conformational and Vibrational Studies of Triclosan. AIP Conf. Proc. 2010, 1203, 1227–1232. 10.1063/1.3322345.

[ref40] Chu C. C.; Williams D. F. The Effect of Gamma Irradiation on the Enzymatic Degradation of Polyglycolic Acid Absorbable Sutures. J. Biomed. Mater. Res. 1983, 17 (6), 1029–1040. 10.1002/jbm.820170612. 6317694

[ref41] Ayala R.; Zhang C.; Yang D.; Hwang Y.; Aung A.; Shroff S. S.; Arce F. T.; Lal R.; Arya G.; Varghese S. Engineering the Cell-Material Interface for Controlling Stem Cell Adhesion, Migration, and Differentiation. Biomaterials 2011, 32 (15), 3700–3711. 10.1016/j.biomaterials.2011.02.004. 21396708

[ref42] Cai S.; Wu C.; Yang W.; Liang W.; Yu H.; Liu L. Recent Advance in Surface Modification for Regulating Cell Adhesion and Behaviors. Nanotechnol. Rev. 2020, 9 (1), 971–989. 10.1515/ntrev-2020-0076.

[ref43] Li H.; Li A.; Zhao Z.; Li M.; Song Y. Heterogeneous Wettability Surfaces: Principle, Construction, and Applications. Small Struct. 2020, 1 (2), 2000028 10.1002/sstr.202000028.

[ref44] Ayyoob M.; Kim Y. J. Effect of Chemical Composition Variant and Oxygen Plasma Treatments on the Wettability of PLGA Thin Films, Synthesized by Direct Copolycondensation. Polymers 2018, 10 (10), 1132 10.3390/polym10101132. 30961057 PMC6403949

[ref45] Almajhdi F. N.; Fouad H.; Khalil K. A.; Awad H. M.; Mohamed S. H. S.; Elsarnagawy T.; Albarrag A. M.; Al-Jassir F. F.; Abdo H. S. In-Vitro Anticancer and Antimicrobial Activities of PLGA/Silver Nanofiber Composites Prepared by Electrospinning. J. Mater. Sci.: Mater. Med. 2014, 25 (4), 1045–1053. 10.1007/s10856-013-5131-y. 24375170

[ref46] Haghighat F.; Ravandi S. A. H. Mechanical Properties and in Vitro Degradation of PLGA Suture Manufactured via Electrospinning. Fibers Polym. 2014, 15 (1), 71–77. 10.1007/s12221-014-0071-9.

[ref47] Chou S.-F.; Woodrow K. A. Relationships between Mechanical Properties and Drug Release from Electrospun Fibers of PCL and PLGA Blends. Fibers Polym. 2017, 65, 724–733. 10.1016/j.jmbbm.2016.09.004. PMC6461716 27756048

[ref48] Manfredini M.; Ferrario S.; Beretta P.; Farronato D.; Poli P. P. Evaluation of Breaking Force of Different Suture Materials Used in Dentistry: An In Vitro Mechanical Comparison. Materials 2022, 15 (3), 1082 10.3390/ma15031082. 35161027 PMC8840186

[ref49] Al-Qattan M. M. Vicryl Rapide versus Vicryl Suture in Skin Closure of the Hand in Children: A Randomized Prospective Study. J. Hand Surg. Eur. Vol. 2005, 30 (1), 90–91. 10.1016/J.JHSB.2004.08.005. 15620501

[ref50] Khiste S. V.; Ranganath V.; Nichani A. S. Evaluation of Tensile Strength of Surgical Synthetic Absorbable Suture Materials: An in Vitro Study. J. Periodontal Implant Sci. 2013, 43 (3), 130–135. 10.5051/jpis.2013.43.3.130. 23837127 PMC3701834

[ref51] Kakisu K.; Matsunaga T.; Kobayakawa S.; Sato T.; Tochikubo T. Development and Efficacy of a Drug-Releasing Soft Contact Lens. Invest. Ophthalmol. Visual Sci. 2013, 54 (4), 2551–2561. 10.1167/iovs.12-10614. 23462746

[ref52] Altun E.; Aydogdu M. O.; Koc F.; Kutlu O.; Gozuacik D.; Yucel S.; Gunduz O. Amoxicillin Loaded Hollow Microparticles in the Treatment of Osteomyelitis Disease Using Single-Nozzle Electrospinning. Bionanosci. 2018, 8 (3), 790–801. 10.1007/s12668-018-0539-y.

[ref53] Shen J.; Burgess D. J. In vitro-in vivo correlation for complex non-oral drug products: Where do we stand?. J. Controlled Release 2015, 219, 644–651. 10.1016/j.jconrel.2015.09.052. PMC4739855 26419305

[ref54] Jacob S.; Nair A. B. An updated overview with simple and practical approach for developing in vitro-in vivo correlation. Drug Dev. Res. 2018, 79 (3), 97–110. 10.1002/ddr.21427. 29697151

[ref55] Rueda-Fernández M.; Melguizo-Rodríguez L.; Costela-Ruiz V. J.; de Luna-Bertos E.; Ruiz C.; Ramos-Torrecillas J.; Illescas-Montes R. Effect of the most common wound antiseptics on human skin fibroblasts. Clin. Exp. Dermatol. 2022, 47 (8), 1543–1549. 10.1111/ced.15235. 35466431 PMC9545306

[ref56] Skubis A.; Gola J.; Sikora B.; Hybiak J.; Paul-Samojedny M.; Mazurek U.; Łos M. J. Impact of Antibiotics on the Proliferation and Differentiation of Human Adipose-Derived Mesenchymal Stem Cells. Int. J. Mol. Sci. 2017, 18 (12), 2522 10.3390/ijms18122522. 29186789 PMC5751125

[ref57] Jin J.; Chen N.; Pan H.; Xie W.; Xu H.; Lei S.; Guo Z.; Ding R.; He Y.; Gao J. Triclosan induces ROS-dependent cell death and autophagy in A375 melanoma cells. Oncol. Lett. 2020, 20 (4), 73 10.3892/ol.2020.11934. 32863906 PMC7436935

[ref58] An J.; Yao W.; Tang W.; Jiang J.; Shang Y. Hormesis Effect of Methyl Triclosan on Cell Proliferation and Migration in Human Hepatocyte L02 Cells. ACS Omega 2021, 6 (29), 18904–18913. 10.1021/acsomega.1c02127. 34337230 PMC8320140

