# Supplementary material for: Pressure-Spun Fibrous Surgical Sutures for Localized Antibacterial Delivery: Development, Characterization, and In Vitro Evaluation
Source: ACS Appl Mater Interfaces. 2023 Sep 20;15(39):45561–73. doi: 10.1021/acsami.3c07956 (PMC10561146; doi:10.1021/acsami.3c07956)
Supplement: Supplementary file 1 — am3c07956_si_001.pdf [file am3c07956_si_001.pdf]

SUPPORTING INFORMATION

# Pressure Spun Fibrous Surgical Sutures for Localised Antibacterial Delivery: Development, Characterisation, and *In Vitro* Evaluation

Esra Altun,[a] Cem Bayram,[b] Merve Gultekinoglu,[b] Rupy Matharu,[c] Angelo Delbusso,[a] Shervanthi Homer-Vanniasinkam,[a] and Mohan Edirisinghe[a*]

[a]*Department of Mechanical Engineering, University College London (UCL), Torrington Place, London WC1E 7JE, United Kingdom*

[b]*Department of Nanotechnology and Nanomedicine, Graduate School of Science and Engineering, Hacettepe University, Ankara 06800, Turkey*

[c]*Department of Civil, Environmental and Geomatic Engineering, University College London, Gower Street, London WC1E 6BT, United Kingdom*

*Email: m.edirisinghe@ucl.ac.uk

## Characterisation Results

DSC was used to evaluate the phase transition behaviour of the components used in the pressure spun fibrous surgical sutures. The thermograms of Tri agent, V-PLGA, 70:30 PP, and 40 TPP fibrous sutures were overlaid in **Figure S1**. The characteristic peaks of each component were observed in the thermograms, indicating their expected behaviour as reported in the literature.[1,2] No additional peaks were detected, suggesting that there were no compatibility issues or unexpected reactions between the components. This confirms the suitability of the selected materials for the fabrication of the pressure spun fibrous surgical sutures and supports their potential practical applications.

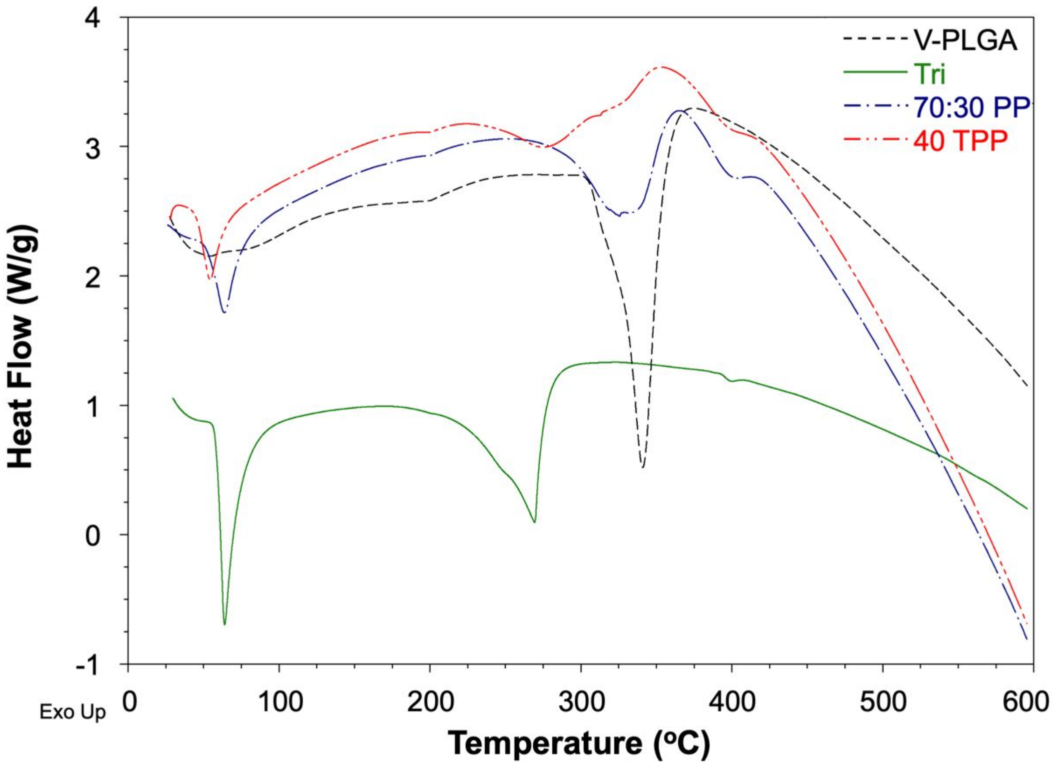


**Figure S1.** DSC curves of Tri agent, V-PLGA, 70:30 PP, and 40 TPP fibrous surgical sutures.

### References


(1) Evrova, O.; Hosseini, V.; Milleret, V.; Palazzolo, G.; Zenobi-Wong, M.; Sulser, T.; Buschmann, J.; Eberli, D. Hybrid Randomly Electrospun Poly(Lactic- *Co* -Glycolic

Acid):Poly(Ethylene Oxide) (PLGA:PEO) Fibrous Scaffolds Enhancing Myoblast Differentiation and Alignment. *ACS Appl. Mater. Interfaces* **2016**, *8 (46)*, 31574–31586.

(2) Lima, C. S. A. de; Varca, G. H. C.; Costa, S. M. da; Ferraz, H. G.; Santos, A. C. da S.; Lopes, P. S.; Costa, S. A. da. Development of Natural Polymeric Microcapsules for Antimicrobial Drug Delivery: Triclosan Loaded Chitosan and Alginate-Based Microcapsules. *Drug Dev. Ind. Pharm.* **2020**, *46 (9)*, 1477–1486.
